# Incentive based emergency demand response effectively reduces peak load during heatwave without harm to vulnerable groups

**DOI:** 10.1038/s41467-023-41970-8

**Published:** 2023-10-04

**Authors:** Zhaohua Wang, Bin Lu, Bo Wang, Yueming (Lucy) Qiu, Han Shi, Bin Zhang, Jingyun Li, Hao Li, Wenhui Zhao

**Affiliations:** 1 School of Management and Economics, Beijing Institute of Technology, Beijing, 100081 China; 2 Research Center for Sustainable Development and Intelligent Decision Making, Beijing, 100081 China; 3 Ministry of Industry and Information Technology Key Lab of Digital Economy and Policy Intelligence, Beijing Institute of Technology, Beijing, 100081 China; 4 School of Public Policy, University of Maryland College Park, College Park, MD 20742 USA; 5 Business School, Sichuan University, Chengdu, 610065 China

**Keywords:** Energy and behaviour, Energy justice, Energy policy

## Abstract

The incentive-based emergency demand response measure serves as an important regulatory tool during energy system operations. However, whether people will sacrifice comfort to respond to it during heatwave and what the effect on heat vulnerable populations will be are still unclear. A large-scale emergency demand response pilot involving 205,129 households was conducted in southwestern China during continuous extreme high temperatures in summer. We found that the incentive-based emergency demand response causes a statistically significant decline in electricity use with no additional financial burden on vulnerable groups. The electricity conservation potential of urban households was higher than that of rural households. Households with children did not respond to the emergency demand response, while the response of households with elderly individuals proved to be more positive. The repeated and frequent implementation of this policy did not result in an attenuation of the regulatory effect. This research can serve as a reference for countries with similar regulated power markets.

## Abstract (web summary)

Co-lead authors Wang, Zhang, Qiu, Lu and their colleagues model an incentive-based emergency demand response to counter heatwaves. The modelled responded leads to the peak load reduction of 7.32% for the covered households, and can achieve a 1.02% peak load reduction when reaching a wide audience with no additional financial burden on vulnerable groups.

## Introduction

The world is experiencing climate change characterized by global warming, and effective ways to reduce greenhouse gas emissions through energy conservation are actively being explored. High temperatures in summer are one of the main causes of peak loads on the power grid^1^, which lead to increases in social energy infrastructure investments and carbon emissions. With the development of the global economy and improvements in electrification processes, electricity consumption has increased sharply^2^. In 2019, the total global final electricity consumption reached 22,848 TWh, of which growth in the residential sector accounted for 26.6%^3^ (Supplementary Fig. 4). The Intergovernmental Panel on Climate Change (IPCC) Sixth Assessment Report also advocated accelerated electrification. The surge in household demand for electricity is an important reason for the double-digit growth in power peak loads and the increased peak-valley difference, leading to the problems associated with an insufficient power supply during peak times; additionally, power rationing occurs from time to time^4^. China and other countries are now faced with the dual pressures of the underutilization of new installed capacity on the power grid supply side and increasing energy consumption on the demand side, creating challenges in matching supply with demand. The traditional approach is to increase the output of the generator set when the load demand is high, but the peak load tends to last for a short time (the peak load duration in a year is only 5% or less; see Supplementary Figs. 5–7). Increasing energy investment is costly, and the installation of new power plants will increase carbon emissions. Therefore, power departments have begun to explore demand response (DR) measures to reduce or delay the peak load on the demand side, especially in the residential sector, to achieve a balance between supply and demand.

DR measures have been widely used worldwide and mainly include two categories: price-based DR measures and incentive-based DR measures (Supplementary Fig. 1)^5,6^. Time-of-use (TOU) is a price-based DR measure that induces households to shift electricity use away from on-peak times by using a fixed rate schedule with more expensive on-peak times^7,8^. Effective pricing is a powerful tool to economically adjust the load curve^9–11^. Pilots carried out in California^12^, Washington D.C.^13^, the Netherlands^14^, and other places have proven that price-based DR measures are stable and provide sizable demand reductions. Users are price responsive, and enabling technologies can improve responses^15^.

Incentive-based DR measures, such as the emergency demand response (EDR), provide extra rewards to reduce demand during on-peak times and are more flexible. Some advanced economies have carried out practical research, and the existing relevant empirical results are mostly institutional reports that prove the effectiveness of the EDR^16–19^. However, large-scale trials have not been carried out, and it is unclear whether the EDR is still effective in countries with a regulated power market such as China.

Furthermore, the effect of DR measures on vulnerable groups, such as low-income households, young children, and elderly individuals have been examined. Low-income households is defined as per capita disposable income below 50% of the national average, which is the threshold used in China’s National Bureau of Statistics definition of low income, and they face pressure to curtail their energy costs, often with negative impacts^20^. The primary strategy adopted by low-income households to cope with financial constraints is to reduce spending, including spending on essentials such as food and energy^21^. Elderly people require a narrower temperature range for health^22^ and are associated with a higher likelihood of mortality in extreme heat events^23–25^. Young children are also negatively affected by household energy insecurity^26,27^. Price-based EDR policy (such as TOU), for households that already struggle with electricity bills, TOU could be detrimental^20,21,28^. Specifically, households suffering from energy poverty are forced to make trade-offs between paying their electricity bills and paying for other necessities such as food and medicine^4,29–31^. Notably, price-based DR measures have been implemented under a deregulated electricity market and with the installation of smart equipment requiring high costs^32^, making it difficult to implement such measures in countries with a partially monopolized power market such as China or on a large scale in low income households. In contrast, incentive-based EDR, compared to TOU, are friendly to these groups since it provides additional rebates which adds no additional financial burden to households who are heat vulnerable. How these groups respond to incentive-based EDR are issues that remain to be clarified before promoting the policy on a national or district scale.

We carried out a series of incentive-based EDR trials involving 205,129 households in southwestern China during continuous extremely high-temperature weather in summer. The trials provided DR enabling technology at no cost to these households, that is, high-speed power line communication (HPLC) meters, which aimed to collect high-frequency electricity use data. We explored voluntary electricity reduction behaviors induced by the EDR, namely the effect of EDR random assignment selection. Then, we further investigated the effect of EDR rebate coverage and heterogeneous effects among vulnerable groups. Finally, we analyzed the sustainability of the EDR effect. This pilot was a large-scale trial in China that analyzed electricity conservation options in an inflexible energy pricing market, and the results can serve as a reference for countries with similar regulated power markets, which have received insufficient attention from previous studies on demand response practices.

## Results

### Effect of the incentive-based EDR on electricity use reductions

**Fig. 1:**
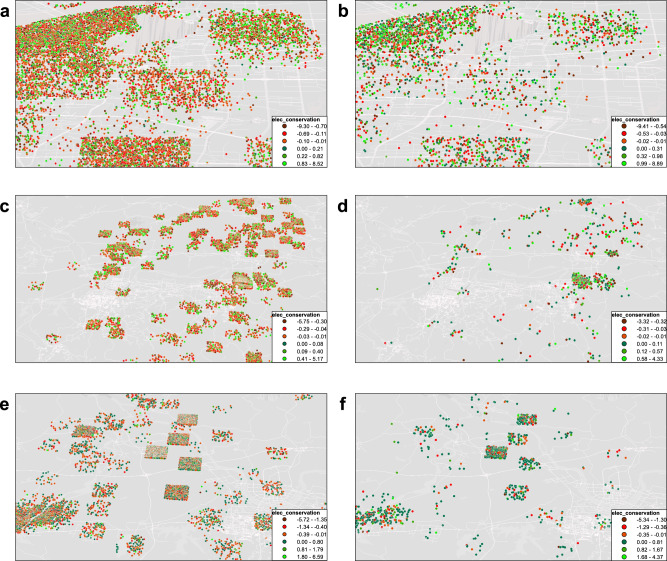
Regional distribution of EDR trials. **a**, **c**, **e** depict the distribution of the no-notification group, and **b**, **d**, **f** represent the distribution of the EDR group. Each subgraph corresponds to a specific trial region (**a**/**b**, **c**/**d**, and **e**/**f** indicate regions with temperature rise (measured on the treatment day relative to the benchmark day) of 0.6 °C, 1.2 °C, and 3 °C, respectively). Electricity conservation indicates the difference in electricity consumption (kWh) between the treatment day and the benchmark day during the EDR period. In each subgraph, individual households are represented by data points, and the color of the point reflects the level of electricity conservation achieved. A greener color indicates a greater amount of electricity saved. Source data for this figure are available on GitHub.

We initiated large-scale incentive-based EDR trials in southwestern China (Fig. 1), drawing 205,129 households by random assignment from pilot areas. When our policy rolls out, all households will receive an EDR invitation message, but not all households receive a rebate, this rebate is only available for those who choose to participate in this EDR program. In our trial design, those who received messages and replied “confirmed” after receiving the messages are called EDR group. Those who received messages but provided no effective feedback are named no-reply group and others who never received any messages are named no-notification group (Supplementary Fig. 3). This random assignment presents us an opportunity to study the effects of EDR random assignment selection and the effect of EDR rebate coverage with a randomized controlled design. It allows us to isolate the causal effect of how effective is the experimental EDR policy at motivating peak reductions through intent-to-treat (ITT) estimate. This random assignment can also be used to study the local average treatment effect (LATE) of EDR rebate coverage on electricity saving without the problem of confounding factors that might otherwise differ between with- and without-reply populations. For households that only received messages and did not respond, we also evaluated the spillover effect of the incentive-based EDR, that is, whether these households would actively reduce their electricity consumption when they were only aware of the EDR message and did not receive monetary rebate (see the details in Supplementary Note 6).

We compare outcomes between the treatment group (EDR group and no-reply group who were randomly selected in the assignment procedure) and the control group (no-notification group). This can be seen as a relevant parameter for gauging the effect of the households winning permission to apply for EDR program. Supplementary Table 9 presents the mean electricity use and descriptive statistics for all groups across the baseline and treatment periods. Our ITT analysis, comparing the outcomes in the treatment and control groups by fitting difference-in-difference regressions, provides an estimate of the causal effect of winning the assignment. Being selected in the random assignment decreases the electricity consumption during our study period by 0.0155 kWh (*p* < 0.001; Table 1) during on-peak times, a 1.02 percent (*p* < 0.001; Supplementary Table 10) reduction relative to the control mean.

**Table 1:** Estimations examining the effect of EDR assignment selection (intent-to-treat test)

	EDR assignment selection		Heterogeneous effect		
	Main effect	Spillover effect	Urban	Children	Elderly
	(1)	(2)	(3)	(4)	(5)
*Treatment×Post×Indicator*			−0.0284*** (0.0036)	−0.0041 (0.0733)	−0.0559** (0.0264)
*Treatment×Post*	−0.0155*** (0.0023)	−0.0024 (0.0016)	0.0063** (0.0027)	−0.0144 (0.0710)	−0.0289* (0.0165)
*Indicator×Post*			−0.0010 (0.0094)	0.1053 (0.0866)	−0.0327 (0.0391)
*Indicator* *×* *Treatment*			0.0113** (0.0044)	−0.0211 (0.0561)	−0.0034 (0.0224)
*Treatment*	−0.0315*** (0.0039)	−0.0226*** (0.0017)	−0.0401*** (0.0048)	0.0016 (0.0527)	−0.0165 (0.0166)
*Post*	−0.0355*** (0.0090)	−0.0372*** (0.0093)	−0.0437*** (0.0122)	−0.1436* (0.0838)	−0.0302 (0.0344)
*Indicator*			−0.1209*** (0.0245)	0.0486 (0.0756)	0.1540*** (0.0440)
Cluster in group	Yes	Yes	Yes	Yes	Yes
Controls	Yes	Yes	Yes	Yes	Yes
Observation	410,258	378,114	410,258	7,466	10,322
F	61.98	79.73	51.94	2.692	11.24
R^2^	0.0085	0.0082	0.0094	0.0077	0.0147

This table reports the estimated coefficients and cluster-robust standard errors (in parentheses). The dependent variable in all columns is electricity usage during on-peak times. Columns (1) and (3–5) estimate the effect of assignment selection comparing the average outcome for households selected in the random assignment (EDR group and no-reply group, which we called assignment winners) to the average outcome for control households (no-notification group those not selected by the assignment) among heterogeneous groups. Column (2) compares the difference between the no-reply group and the no-notification group. The standard errors are clustered at the household-group level. Significance is at ***p < 0.01, **p < 0.05, *p < 0.1.

In the pilot area, we have no evidence that the EDR had spillover effects. Compared to the no-notification group, the electricity usage of the no-reply group did not change significantly (coef. = *−*0.0024; *p* = 0.149; Table 1) during on-peak times. This means that a mere message notification did not lead to a reduction in electricity usage in this trial.

**Fig. 2:**
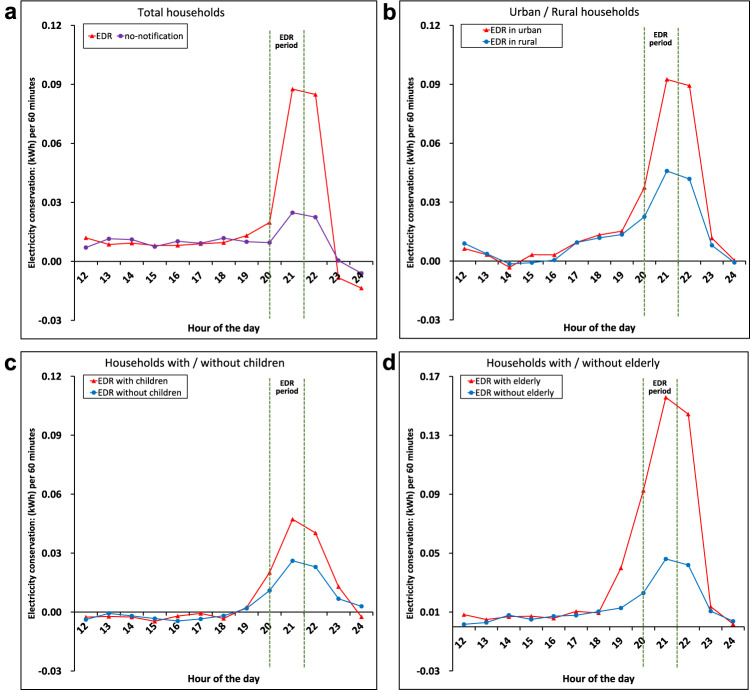
Effects of the incentive-based EDR on electricity usage among heterogeneous groups. **a**–**d** depict the electricity conservation of the EDR group and no-notification group among heterogeneous groups. The ordinate represents the difference in electricity usage (measured in kWh) between the benchmark day and treatment day. A positive value indicates a reduction in electricity use. **a** represents the overall effect, with the red line representing the EDR group, and the purple line representing the no-notification group. **b**, **c**, **d** demonstrate the heterogeneous effects on urban households, households with children, households with elderly individuals and the control group, respectively. Source data for this figure are available on GitHub.

The intent-to-treat provides an estimate of the net impact of expanding access to EDR, which may lead to conservative effects of the rebate coverage. Not everyone selected by the random assignment enrolled in the EDR program, some did not respond to apply for coverage. We show visual evidence from the raw data in Fig. 2, which plots the electricity conservation for various populations during the EDR trials. The figure indicates that usage in the pretreatment hours was essentially the same for all groups on the benchmark day and treatment day (see the complete figure in Supplementary Fig. 12). EDR group had more electricity conservation on average than the no-notification group during our trial period. EDR rebate coverage increases the probability of saving behavior by 3.68 times for 0.1297 kWh relative to the control mean of 0.0352 kWh (Supplementary Table 9).

To estimate the causal effect of EDR rebate coverage, we use a standard instrumental-variable approach with assignment selection as an instrument for EDR rebate coverage. This estimates the local average treatment effect capturing the causal effect of EDR for those who were covered because of the random assignment, under the assumption that winning the assignment only impacts the outcomes studied through EDR rebate coverage. It could be seen as the relevant parameter for evaluating the causal effect of EDR rebate for those who participated in the EDR program because of receiving the message. Imperfect (and non-random) take-up of EDR among those selected in the random assignment reduces statistical power, but does not confound the causal interpretation of the effect of EDR. As shown in Table 2, EDR rebate coverage decreases electricity consumption by 0.1145 kWh (*p* < 0.001; Table 2) during on-peak times, a 7.32 percent (*p* < 0.001; Supplementary Table 11) reduction relative to the control mean of 0.04 kWh. In addition, we obtained robust results by using matching methods to solve the problem of parallel trends between the treatment group and the control group. A series of robustness tests, including the Heckman two-step method test and placebo test, have also confirmed this conclusion (see Supplementary Note 6 for details).

**Table 2:** Estimations examining the effects of EDR rebate coverage (IV two-stage approach and DTW matching method)

	EDR rebate coverage (IV two-stage)				EDR rebate coverage (DTW-matching)			
	Total	Urban	Children	Elderly	Total	Urban	Children	Elderly
	(1)	(2)	(3)	(4)	(5)	(6)	(7)	(8)
*Treatment* *×* *Post* *×* *Indicator*		−0.0545*** (0.0190)	−0.0138 (0.1827)	−0.1638** (0.0737)		−0.0699*** (0.0184)	−0.0223 (0.1854)	−0.1677** (0.0762)
*Treatment* *×* *Post*	−0.1145*** (0.0302)	−0.0468*** (0.0169)	−0.0500 (0.1745)	−0.1070* (0.0609)	−0.0928*** (0.0121)	−0.0303* (0.0180)	−0.0438 (0.1759)	−0.1136** (0.0546)
*Indicator* *×* *Post*		0.0312*** (0.0055)	0.1059* (0.0630)	−0.0320 (0.0294)		0.0039 (0.0096)	0.1073 (0.0869)	−0.0333 (0.0392)
*Indicator* *×* *Treatment*						0.0365 (0.0223)	−0.1564 (0.1705)	−0.0243 (0.0720)
*Treatment*					−0.0816*** (0.0205)	−0.1113*** (0.0303)	0.1311 (0.1568)	−0.0139 (0.0589)
*Post*	0.0471** (0.0226)	−0.0091 (0.0200)	−0.2796* (0.1492)	−0.1325 (0.1163)	−0.0339*** (0.0093)	−0.0472*** (0.0127)	−0.1488* (0.0852)	−0.0316 (0.0348)
*Indicator*						−0.1311*** (0.0248)	0.0427 (0.0775)	0.1534*** (0.0445)
Cluster in group	No	No	No	No	Yes	Yes	Yes	Yes
Controls	Yes	Yes	Yes	Yes	Yes	Yes	Yes	Yes
Observation	410,258	410,258	7,466	10,322	222,554	222,554	4124	5814
R^2^	0.0033	0.0035	0.0080	0.0222	0.0111	0.0121	0.0209	0.0072

This table reports the estimated coefficients and cluster-robust standard errors (in parentheses). The dependent variable in all columns is electricity usage during on-peak times. Columns (1–4) use a standard instrumental-variable approach with assignment selection as an instrument to estimate EDR rebate coverage among heterogeneous groups. Column (5-8) compares the difference between the EDR group and the no-notification group by using matching methods. The standard errors are clustered at the household-group level. Significance is at ***p < 0.01, **p < 0.05, *p < 0.1.

### Discrepancy among vulnerable groups in sensitivities to the EDR

**Fig. 3:**
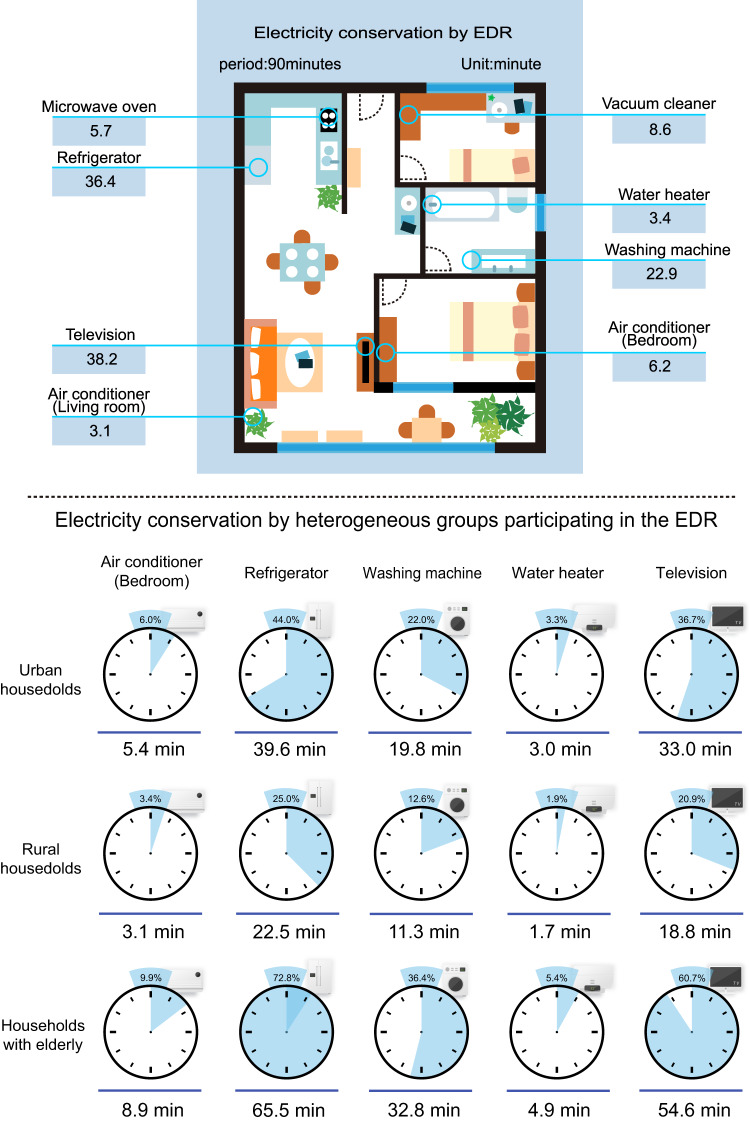
Electricity conservation resulting from the incentive-based EDR in terms of the number of minutes that home appliances can be turned off. The data in the layout of the house above the horizontal line indicates the specific time intervals corresponding to the electricity saved by the EDR in terms of home appliances. Each subgraph below the horizontal line represents the duration in minutes that home appliances need to be turned off to achieve the average electricity reduction in urban, rural, and elderly households participating in the EDR. Each circle encompasses 60 min, and the value above each circle represents the percentage of time that home appliances are turned off during on-peak periods. The power of home appliances comes from the smart sockets that we installed in 15 households, and the behaviors that households may carry out are calculated based on the average electricity conservation of each group. Source data for this figure are available on GitHub.

We also examine how the effects of EDR program on electricity conservation differ among heterogeneous groups. Tables 1 and 2 report the effect of assignment selection and EDR rebate coverage, which confirm the more conservative effect of intention-to-treat. For EDR rebate coverage, urban households participating in the EDR program saved 0.0545 kWh (*p* < 0.001; Table 2; 2.19% peak load reduction, Supplementary Table 11) more than rural counterparts. In addition, considering that electricity use was expected to be driven by cooling needs during heatwave, our main estimation strategy followed the same logic as a standard difference-in-difference-in-difference (DDD) strategy to examine whether the EDR still worked when the temperature increased among heterogeneous groups (more details in Supplementary Note 4). We find that EDR weakens the effect of temperature on electricity consumption (Supplementary Tables 3, 7–8), which has the same performance in urban and rural households. Contrary to expectations, compared to households without children counterparts, households with children participating in the EDR saw no significant effect (coef. = *−*0.0138*, p* = 0.940; Table 2). Compared to households without elderly individuals counterparts, households with elderly individuals participating in the EDR saw a larger effect and saved 0.1638 kWh (*p* = 0.026; Table 2). We also obtained robust results by using a DID approach within the heterogeneous populations (Supplementary Table 12). According to the smart socket data collected from some participants (Supplementary Note 10), we show the electricity conservation behaviors that the households may carry out in Fig. 3.

### Sustainability of the incentive-based EDR effect

A central question for policymakers designing EDR is whether appealing to intrinsic and extrinsic motivations can generate persistent effects on electricity conservation behavior^33^. After all, EDR policies are meant to be implemented repeatedly for the long term. To estimate the sustainability of the incentive-based EDR effect, we repeated our interventions over six treatment days in the summer (Supplementary Note 8). We examined the sustainability of the treatment effect through ordinary least squares (OLS) estimations with treatment phases (see “Methods”). As shown in Table 3, the EDR produced much more persistent effects with repeated stimuli. Column (1) indicates that the more times the households participating in the EDR, the greater the amount of electricity that was saved during on-peak times in general (coef. = 0.0406; *p* < 0.001). In our trial, the treatment effect was the largest in the fifth phase (coef. = 0.1326; *p* < 0.001). Columns (5) and (7) indicate that urban households (coef. = 0.0407; *p* < 0.001) were slightly more affected by the number of times participating than rural households (coef. = 0.0381; *p* < 0.001). We also need to be cautious about the treatment effect with almost no decay results, which may be related to the limited number of trials.

**Table 3:** Estimations examining the sustainability of the incentive-based EDR effect

	EDR effect				Heterogeneous effect			
	EDR rebate coverage		EDR spillover effect		Rural		Urban	
	(1)	(2)	(3)	(4)	(5)	(6)	(7)	(8)
*Treat_cnt*	0.0406*** (0.0031)		0.0095*** (0.0010)		0.0381*** (0.0049)		0.0407*** (0.0032)	
*1st phase*		0.0822*** (0.0161)		0.0131 (0.0242)		0.0797** (0.0375)		0.0829*** (0.0167)
*2nd phase*		0.0952*** (0.0086)		0.0224*** (0.0034)		0.0852*** (0.0133)		0.0970*** (0.0091)
*3rd phase*		0.1159*** (0.0160)		0.0230*** (0.0069)		0.1066** (0.0422)		0.1173*** (0.0159)
*4th phase*		0.1295*** (0.0275)		0.0355*** (0.0129)		0.0919** (0.0351)		0.1310*** (0.0345)
*5th phase*		0.1326*** (0.0336)		0.0332*** (0.0116)		0.0969*** (0.0367)		0.1246*** (0.0353)
Cluster in group	Yes	Yes	Yes	Yes	Yes	Yes	Yes	Yes
Controls	Yes	Yes	Yes	Yes	Yes	Yes	Yes	Yes
Observation	249,953	249,953	394,531	394,531	56,366	56,366	172,105	172,105
F	59.65	49.49	59.04	49.02	24.20	19.94	48.27	38.65
R^2^	0.014	0.014	0.0119	0.0119	0.010	0.010	0.015	0.015

This table reports the estimated coefficients and cluster-robust standard errors (in parentheses). The dependent variable in all columns is electricity conservation during on-peak times. We include household fixed effects and time fixed effects. The standard errors are clustered at the household-group level to adjust for serial correlation. Significance is at ***p < 0.01, **p < 0.05.

Our findings on sustainability have four key policy implications, particularly for policymakers aiming to generate persistent policy impacts over repeated interventions. The incentive-based EDR is likely to produce a sizable effect on households that have participated multiple times, and after being stimulated many times, its spillover effect (after the 2nd phase) shows slight electricity conservation. Repeated interventions do not result in an attenuation of electricity conservation, and monetary incentives induce larger treatment effects.

## Discussion

We conducted the EDR pilot involving 205,129 households from southwestern China during heatwave. We estimated the intent-to-treat effects of the incentive-based EDR during on-peak times and isolate the causal effect of EDR rebate coverage on electricity conservation among vulnerable groups using the random assignment of the study design. In addition, we examined the sustainability of the incentive-based EDR effect.

The results suggest that incentive-based EDR decreases peak-loads considerably during heatwave. In 2019, the National Development and Reform Commission and National Energy Administration of China requires all provinces to form a demand response capacity to reach 3% of the annual maximum electricity load to deal with summer energy spikes. This is a crucial task for China every summer especially with the accelerating electrification process after 2020. Our EDR program can adjust the peak load reduction of 7.32% for the covered households, and can achieve a 1.02% peak load reduction even when reaching a wide audience (those receiving the EDR message). We have examined varying treatment effects across different electricity prices and the results show that households with higher electricity prices tend to respond more actively to EDR (Supplementary Note 11). This is from the residential sector alone, which has reduced orderly power consumption pressure in industries. This is meaningful from the perspective of system management.

From a cost-benefit point of view, the program helps slow down the need to build new power plants to meet short-term spikes in summer and reduce the electricity consumption loss proportion from the industrial sector. Compare to power plant construction cost and corporate profits and tax, our cost is minimal. Besides, our EDR program only pays a rebate for households who choose to participate. Our findings speak to the rebate cost of expanding incentive-based EDR, as well as its net effect on the peak load reduction, and may thus be a useful input for informed decision-making balancing the costs and benefits of peak load reduction.

The policy is intrinsically different from those based on TOU rates, which are a common DR measure that might worsen the heating or eating dilemma of vulnerable groups, and that can also be inoperable in countries with a regulated power market such as China. Incentive-based EDR can promote voluntary reductions in electricity usage with extra incentives, which is effective in shifting demand away from on-peak times during heatwave and has no extra financial burdens related to trade-off pressures within vulnerable groups. Furthermore, EDR becomes more effective after repeated stimuli. As a supplementary DR measure, the EDR is one of the few treatment options to have undergone multiple trials. It provides a practical, economical and flexible solution for relieving the contradictions in seasonal power supply and demand and for reducing carbon emissions in the context of global warming, especially for countries with a regulated power market.

Heterogeneity analysis indicates that there are differences in EDR effects among vulnerable groups, which highlights the importance of considering specific subpopulations in the design and rollout of EDR policies to avoid creating new inequities. We found that the electricity saving potential of urban households is higher than that of rural households, which may be related to household income and the number of home appliances^34^. Compared with rural low-income households, urban households have a higher disposable income, larger house areas, and more home appliances^35^, leading to more demand for electricity use and thus a greater electricity conservation potential (The per capita disposable income of rural households in 2018 was 14,617 yuan, only 37% of that of urban residents, and 20% of them were even <3666 yuan.). In addition, the efficiency of home appliances and electronic devices is critical for electricity conservation, and high-income households have the ability to purchase smart devices and to install home power management systems that will help them save more electricity^36^. Both urban and rural households can reduce their electricity usage under stimulation by economic incentives. Considering that some low-income households can afford only light bulbs, even if they were willing to participate in the EDR, the electricity conservation potential was very limited. In the case of high efficiency and limited costs, power departments might be more willing to carry out EDR in urban areas because the electricity conservation of urban households is greater than that of rural households (Tables 1 and 2). The money used to incentivize EDR could be disproportionately distributed to urban households, which find it easier to reduce their electricity use. These types of transfer payments may be unfavorable for low-income groups. We also conducted a sub-group regression analysis to estimate the differences between the self-owned houses and tenants. Our findings indicate that EDR has a more significant effect on owner’s electricity savings behavior during peak hours compared to tenants (Supplementary Note 12).

For households with children, parents tend to provide their children with a comfortable living environment because children are young and have weak physical resistance to fluctuations in temperature^37^. In addition, Chinese children have a considerable amount of homework in summer, and parents are willing to provide a cool study environment for children during heatwave^38^. The comfort and learning efficiency of children outweigh the limited financial incentives to reduce electricity usage. The fact that the EDR had a significant effect on elderly households could be due to the thriftiness of older generations in Asian countries. According to the theory of continuity, a person lives and develops in specific environmental conditions, and the lifestyle formed has continuity, which guides the activities of elderly individuals. Since elderly Asian individuals have a frugal lifestyle^39^, they are more inclined to save electricity when stimulated by economic incentives. However, they can choose to participate or give up doing so voluntarily based on their own conditions after evaluating their comfort and benefits. Therefore, we still need to consider that the potential threat of elderly people overheating or suffering other health hazards out of thrift is probably worse.

Economic incentives can generate persistent impacts on electricity reduction^40^. There are two potential mechanisms. One is related to “learning by doing”^41^. In the beginning, households might not know how to reduce their electricity use, but as trials are carried out repeatedly, they begin to plan ahead and engage in practices such as turning off the air conditioner, preheating the water heater, and refrigerating the room to a lower temperature in advance (empirical evidence from Supplementary Note 10). The other possibility might be related to investments in smart energy-efficient appliances^42^. We do not expect such investments to be made extensively anytime soon, but we can expect them to become more commonplace over the long run.

## Methods

### Participants

The data are from households that participated in the EDR pilot administered by researchers and power departments in southwestern China. We randomly assigned permission to apply for the EDR trial. We sent EDR messages to all households except the no-notification group (those not selected by the assignment); therefore, only assignment winners (the EDR group and the no-reply group) were selected in the assignment households. Those in the EDR group who replied to the message to confirm participation were seen as targeted participants. Those in the no-reply group who did not reply or replied “no” were seen as untargeted participants. Those in the no-notification group who did not receive any treatment were as seen as the pure comparision group. We preprocessed the electricity use data (vacant homes that were always at 0 kWh were removed) and deleted the households with missing values caused by collection and transmission by HPLC smart meters. The final sample retained 205,129 households, including 53,129 rural households and 152,000 urban households. The survey sample retained 7774 households, including 3203 households with children and 2991 households with elderly individuals.

### Procedures

#### EDR rebate program

We carried out this research with the state grid. We first adopted the clustered randomization method to randomly select the region of the EDR trial (divided by communities, a total of 205,129 households were selected) and then installed HPLC smart meters, which can collect electricity consumption data at 15-minute intervals. We used the exact same procedures to conduct six EDR trials based on monetary rebate in southwestern China from July 18, 2019, to August 21, 2019 (Supplementary Table 1).

The peak load hours usually appear between 8 and 9:30 pm in southern China in July–August (Supplementary Fig. 8). The power infrastructure is sized based on the system peak load. Thus, reducing the peak hour demand is more critical than reducing the demand in other hours, which helps improve grid stability and lower the likelihood of blackouts. In order to reduce peak load and explore the energy-saving potential of households during the on-peak times, we set the period for emergency demand response trial to 8–9:30 pm. On the morning of the treatment day, we sent a phone message (see the English translation of the recruitment message in Supplementary Note 2) to inform households that we were going to carry out the EDR trial from 8 pm to 9.30 pm that day. Of these households, those that replied “yes” confirmed their participation. Those that did not reply or that replied with an irrelevant message were not considered to be treated. Similar to previous field experiments in electricity demand^13,43,44^, we still need to solve some potential endogeneity problems, although the random assignment of the treatment guarantees the internal validity of the experiment. To explore the external validity of our sample, we collected data from a random sample of the population in the corresponding geographical area. We analyzed the observables between our sample and the random sample. Three pieces of sample data were collected from the households as follows. For sample A, the EDR group, the 16,072 households in this group installed an advanced HPLC meter and received EDR messages. Those in this group confirmed their participation. For sample B, the no-reply group, the 93,852 households in this group installed an advanced HPLC meter and received EDR messages. Those in this group did not confirm their participation. For sample C, the no-notification group, the 95,205 households in this group installed an advanced HPLC meter. This group received no other treatment (Supplementary Fig. 3).

#### Economic incentives

For households participating in the EDR trial, if electricity use during the declared on-peak times (8 pm to 9.30 pm on the treatment day) was 1 kWh lower than that during the same period on the day before (benchmark day), the saved electricity generated a cash incentive of $0.143/kWh (Equivalent to RMB as ¥1), given as bill credits. The pilot provinces in Southwest China adopt the increasing block price for residents (Supplementary Fig. 19) and the incentive is greater than the block prices and average electricity prices. We examine varying treatment effects across varying electricity prices and the results show that households with higher electricity prices tend to respond more actively to EDR, resulting in greater electricity savings during peak hours. In addition, we find that EDR has more substantial effects on households with higher marginal electricity prices, which is consistent with the conclusion in heterogeneous treatment effect analysis. The detailed analysis can be found in Supplementary Note 11.

#### Different types of housing occupancy

In the pilot study, we obtained the renter and owner information from the survey. Among those who returned valid surveys, 6462 households are self-owned and 1312 are tenants. Whether people live in their own house may lead to differences when they are choosing to participate (or not) in the EDR pilot. We adopt an instrumental variable two-stage approach to estimate the effect of the treatment—not just assignment to treatment—that can account for noncompliance. To adjust for noncompliance, one can use the random assignment to treatment as an instrument for treatment receipt since the initial assignment was random. We estimate the effects of EDR rebate coverage by fitting two-stage least squares regressions on the subsample of survey respondents who reported self-owned and tenant information. Our findings indicate that EDR has a more significant effect on owner’s electricity savings behavior during peak hours compared to tenants (coef. = −0.1472, *p* = 0.028, Supplementary Table 25). The detailed analysis can be found in Supplementary Note 12.

#### Other data

We also collected data on the historical monthly electricity use of the households and hourly meteorological data from nearby monitoring stations, source data can be found at Data availability section. Importantly, there were persistent high temperatures in the pilot area, with daily maximum temperatures above 35 °C during the trial period.

### Survey

A survey investigating the demographic characteristics of the households and assessing their electricity consumption habits was administered between July and December 2019 (see Supplementary Note 13). We conducted this survey mainly through online forms, and the scope of the survey was randomly selected households in the EDR pilot. After the surveys were collected, we tested the validity of the surveys through a rigorous screening process. For the full sample (*n* = 7774), 41.2% of the participating households had children, and 38.5% of the participating households had elderly individuals.

### Analyses

#### Matching

EDR rebate program can be seen as an exogenous shock; however, the selection of the treatment group might not be completely random. For example, some low-income households, households with energy conservation potential, or households that are more sensitive to monetary incentives might be more inclined to participate in the EDR, which might result in self-selection bias. First, we apply household-level fixed effects to control for time-invariant and unobserved characteristics, such as income, occupation, energy preference, and money sensitivity. The fixed effects model can only partially control for endogeneity problems, and the correction effect is very limited if the omitted variables contain time-varying and unobservable factors. Second, we use a matching method. It is difficult for traditional matching methods (such as propensity score matching) to capture all of the factors that affect the electricity use behavior during the declared on-peak times through the covariates. We thus adopt a dynamic time warping (DTW)-based matching method to control for the parallel trend between the treatment and control groups^45^. This method integrates the households’ 15-min high-frequency and monthly low-frequency electricity use data on a microscale. From the perspective of behavioral results, we believe that the historical electricity use fluctuation trend contains known, unknown, or difficult-to-measure variables with traditional methods that affect the willingness to participate in the EDR. The households with similar electricity use patterns in the long term (36 months) and short term (15 min) before the trial should be comparable. However, there might be other self-selection bias based on other unobserved factors other than that can be captured by long- and short-term pattern (although the likelihood is low since the parallel trends assumption is satisfied, Supplementary Fig. 15). We apply this method to divide all samples into different types of electricity use patterns to ensure that the potential results are randomly distributed within the group (for details, see Supplementary Note 6).

#### Difference-in-difference

As a classic method of causal inference, the DID method has been widely used^46–49^. The DID analysis of the effect of the EDR is defined as follows (Eq. (1)):

1 $${{Elec}}_{{it}}={{{{{{\rm{\beta }}}}}}}_{0}+{{{{{{\rm{\beta }}}}}}}_{1}\left({{Treatment}}_{i}\times {{Post}}_{t}\right)+{{{{{{\rm{\beta }}}}}}}_{2}{{Treatment}}_{i}+{{{{{{\rm{\beta }}}}}}}_{3}{{Post}}_{t}+{X}_{{it}}+{{{{{{\rm{\varepsilon }}}}}}}_{{it}}$$

where ${{Elec}}_{{it}}$ is the electricity usage of households during the declared on-peak times. ${{Treatment}}_{i}$ is an indicator of ${{Assignment}}_{i}$, ${{EDR}}_{i}$ and ${{Message}}_{i}$ when estimating the effect of assignment selection, EDR rebate coverage and the spillover effect, respectively. ${{Assignment}}_{i}$ is a dichotomous variable set to 1 if the household was assigned to the EDR program and 0 for others. ${{EDR}}_{i}$ is a dichotomous variable set to 1 if the household participated in the EDR program and 0 for others. ${M{essage}}_{i}$ is a dichotomous variable set to 1 if the household only received the EDR message and 0 if the household did not receive the message. ${P{ost}}_{t}$ is a dichotomous variable set to 1 if the date was the treatment day and 0 for the benchmark day. ${{Treatment}}_{i}\times {{Post}}_{t}$ controls for the effect on electricity use due to the EDR program during on-peak times. ${\beta }_{1}$ is the DID estimate of the treatment effect. The term ${X}_{{it}}$ represents the control variables, including wind direction, wind speed, relative humidity, pressure, the average monthly electricity use of households and other factors related to household characteristics. ${\varepsilon }_{{it}}$ is the idiosyncratic error term. In addition, we cluster standard errors at the cluster-group level to allow for arbitrary serial correlation and correlation across households within the cluster groups by using DTW matching method. We validate the parallel trends assumption in Supplementary Note 9.

#### Difference-in-difference-in-difference

To examine the heterogeneous effects of the EDR program, we built the DDD model defined as follows (Eq. (2):

2 $${{Elec}}_{{it}}=	{{{{{{\rm{\beta }}}}}}}_{0}+{{{{{{\rm{\beta }}}}}}}_{1}\left({{Treatment}}_{i}\times {{Post}}_{t}\times {Indicator}_{i}\right)+{{{{{{\rm{\beta }}}}}}}_{2}\left({{Treatment}}_{i}\times {{Post}}_{t}\right) \\ 	+{{{{{{\rm{\beta }}}}}}}_{3}\left({{Treatment}}_{i}\times {Indicator}_{i}\right)+{{{{{{\rm{\beta }}}}}}}_{4}\left({{Post}}_{t}\times {{Indicator}}_{i}\right) \\ 	+{{{{{{\rm{\beta }}}}}}}_{5}{{Treatment}}_{i}+{{{{{{\rm{\beta }}}}}}}_{6}{{Post}}_{t}+{{{{{{\rm{\beta }}}}}}}_{7}{{Indicator}}_{i}+{X}_{{it}}+{{{{{{\rm{\varepsilon }}}}}}}_{{it}}$$

where ${{Indic}a{tor}}_{i}$ is a dichotomous variable for households set to 1 if the household is a certain type of household (an urban household, a household with children, a household with elderly individuals) and 0 for other households. ${{Indic}a{tor}}_{i}\times {{Post}}_{t}$ controls for the differences experienced during on-peak times by heterogeneous households regardless of the EDR program. ${{Indic}a{tor}}_{i}\times {{Treatment}}_{i}$ controls for the differences in heterogeneous households for the EDR regardless of whether the trial had begun or not. The term of interest is ${{Treatment}}_{i}\times {{Post}}_{t}\times {{Indic}a{tor}}_{i}$, which indicates the effect of the EDR during on-peak times among heterogeneous households. All other variables are as defined in Eq. (1).

#### Instrumental variable two-stage approach test

We adopt an instrumental variable two-stage approach to estimate the effect of the treatment— not just assignment to treatment—that must account for noncompliance. To adjust for noncompliance, one can use assignment to treatment as an instrument for treatment receipt since the initial assignment was random. We estimate the effects of EDR by fitting two-stage least squares regressions (with assignment selection as an instrument for EDR coverage) and estimating the local average treatment effect of EDR coverage. We model this as follows (Eq. (3)):

3 $${Ele}{c}_{{it}}={{{{{{\rm{\pi }}}}}}}_{0}+{{{{{{\rm{\pi }}}}}}}_{1}{{Treatment}}_{{it}}+{X}_{{it}}+{{{{{{\rm{\alpha }}}}}}}_{i}+{u}_{{it}}$$

where ${Treatmen}{t}_{{it}}$ is defined as participating in the EDR trial during the study period. All other variables are as defined in Eq. (1). We estimate Eq. (3) by instrumental variable regression using the following first-stage equation:

4 $${Treatmen}{t}_{{it}}={{{{{{\rm{\beta }}}}}}}_{0}+{{{{{{\rm{\beta }}}}}}}_{1}{{IV}{\_}{assignment}}_{{it}}+{X}_{{it}}+{{{{{{\rm{\alpha }}}}}}}_{i}+{v}_{{it}}$$

in which the excluded instrument is the variable ${IV}\_a{ssignment}$ that is a dichotomous variable set to 1 if the household was assigned to the EDR and 0 for others.

We interpret the coefficient on ${Treatmen}{t}_{{it}}$ from the instrumental variable estimation as the local average treatment effect of EDR. In other words, our estimate of ${{{{{{\rm{\pi }}}}}}}_{1}$ identifies the causal effect of EDR among the subset of households who participated in the EDR upon winning the assignment but who would not participate in EDR without winning the assignment (i.e., the compliers).

#### Sustainability of the incentive-based EDR effect

We examined the sustainability of the treatment effects through OLS estimations with the treatment phases.

5 $${{Elec}{\_}{conservation}}_{{it}}={\delta }_{0}+{\delta }_{1}{{Treat}{\_}{cnt}}_{{it}}+\gamma {X}_{{it}}+{\alpha }_{i}+{\lambda }_{t}+{\varepsilon }_{{it}}$$

6 $${{Elec}{\_}{conservation}}_{{it}}=\mathop{\sum}\limits_{t\in T}\left({\beta }_{t}{{EDR}}_{{it}}+{\varphi }_{t}{{Spillover}}_{{it}}\right)+{\alpha }_{i}+{\lambda }_{t}+{\varepsilon }_{{it}}$$

where ${{Elec\_conservation}}_{{it}}$ is the electricity conservation of households during the declared on-peak times. ${{Treat}{\_cnt}}_{{it}}$ refers to the cumulative number of times participating in the EDR of household *i* in phase *t*. ${\beta }_{t}$ and ${\varphi }_{t}$ are the effect of EDR rebate coverage and the spillover effect in treatment phase *t*, respectively. The term ${\alpha }_{i}$ is individual fixed effects, and it captures the time-invariant characteristics of household *i*. The term ${\lambda }_{t}$ is time fixed effects, and it captures the time-variant characteristics of phase *t*.

#### Robustness test. Parallel trend test

An important hypothesis for the DID method is that the parallel trend between the treatment and control groups is consistent and that there is no systemic difference over time. We selected the electricity use data of the same declared on-peak times for six continuous days (8 pm–9.30 pm from August 15, 2019, to August 20, 2019) to generate interaction terms using time dummy variables and treatment group dummy variables. The interaction terms were used as explanatory variables for the regression, and the coefficients reflect the difference between the treatment and control groups. We found that the EDR had a significant effect only on the treatment day, and there were no systematic differences between the treatment and control groups before the trial (Supplementary Fig. 15).

#### Heckman two-step method test

We collected factors that may affect households’ participation in the EDR through a survey. In the first stage, the selection equation was constructed, and some factors causing the participants’ potential motivation were selected as the exclusive constraint variables. Other factors that may affect the response were controlled for, such as the house area, the number of family members, whether the occupation is in the energy and environmental protection industries, income, electric vehicles, the region, the community type, the number of air conditioners, the number of major appliances, and the average monthly electricity use. In the second stage, the inverse Mills ratio was added for regression. We found that Heckman’s coefficient was significant (coef. = −0.1239, *p* = 0.027; Supplementary Table 16), and the coefficients of other similar methods were also significant (OLS coef. = −0.0844, *p* = 0.060; HeckMLE coef. = −0.1627, *p* = 0.033; Heck2SLS coef. = −0.1626, *p* = 0.033). These results are consistent with the conclusion of the paper (for more details, see Supplementary Note 6).

#### Placebo test

We performed a placebo test to conduct a counterfactual test by changing the implementation time of the EDR. Specifically, we set up hypothetical treatment and control groups and a hypothetical EDR trial implementation time. We selected the electricity use data of the same households on non-EDR days (August 15, 2019, and August 16, 2019, that is, assuming the EDR was implemented some days in advance). We took the households that actually participated in the EDR as the hypothetical treatment group, and the remaining households were used as the hypothetical control group. The regression results (Supplementary Tables 17–18) show that the key estimated coefficients in each group were not significant, which means that following the removal of the EDR trial, there were no systematic differences in the changes in electricity use between the treatment and control groups. This finding proves that our previous estimation results are robust.

### Inclusion and diversity statement

We value diversity in our research and strive for a culture of inclusion. We pledge to cultivate an environment and culture that promotes inclusion and values the respectful participation of all individuals who help advance the mission.

### Reporting summary

Further information on research design is available in the Nature Portfolio Reporting Summary linked to this article.

### Supplementary information

Supplementary Information

Reporting Summary

### Source data

Source Data

## Data Availability

The individual electricity consumption data were obtained from the State Grid through the high-speed power line communication (HPLC) smart meters. We are restricted by a non-disclosure agreement and cannot share the individual data publicly, but information about the aggregated statistics generated during the study can be found on GitHub at https://github.com/BinLu-leo/Emergency-Demand-Response-effect. Other data used for this study are all retrieved from publicly available sources and the sources for each variable can be found in the final compiled datasets (excluding the individual electricity consumption data) and source data can be found on GitHub at https://github.com/BinLu-leo/Emergency-Demand-Response-effect. Source data are provided with this paper.

## Code availability

All code used to process data and generate the figures, tables, and results in this study are publicly available on GitHub at https://github.com/BinLu-leo/Emergency-Demand-Response-effect.
