# Supplementary material for: Incentive based emergency demand response effectively reduces peak load during heatwave without harm to vulnerable groups
Source: Nat Commun. 2023 Oct 4;14:6202. doi: 10.1038/s41467-023-41970-8 (PMC10550920; doi:10.1038/s41467-023-41970-8)
Supplement: Supplementary file 1 — Supplementary Information [file 41467_2023_41970_MOESM1_ESM.pdf]

Supplementary Information

Incentive based emergency demand response effectively and vulnerable friendly reduce peak load during heatwave

Table of Contents

Supplementary Note 1. Brief Introduction to the Demand Response (DR) 3

Supplementary Note 2. Experiment Description 5

Note 2.1 Procedure 5

Note 2.2 Sample Description 7

Supplementary Note 3. Global Final Electricity Consumption and the Temporal Load in the EU and Some Neighboring Countries 8

Supplementary Note 4. Relationship between Temperature and Electricity Use 11

Note 4.1 Estimates of households' electricity use during hot spells 11

Note 4.2 Estimate the effect of the EDR when the temperature rises 17

Supplementary Note 5. Descriptive Statistics 19

Supplementary Note 6. Methods and Endogeneity Problems 21

Note 6.1 Analytic specifications: Effect of random assignment selection (intent to treat) 21

Note 6.2 Analytic specifications: Effect of EDR rebate coverage (instrumental variable two-stage approach) 21

Note 6.3 Analytic specifications: Other method to solve endogenous problems 22

Supplementary Note 7. Supplementary Tables of Detailed Results 26

Supplementary Note 8. Sustainability of the Incentive-Based EDR Effect 30

Supplementary Note 9. Robustness test 31

Note 9.1 parallel trend test 31

Note 9.2 Heckman two-step method test 31

Note 9.3 Placebo test 35

Supplementary Note 10. Conversion standard for power and electricity use of household appliances 36

Supplementary Note 11. Heterogeneous treatment effect analysis across different electricity price 39

Supplementary Note 12. Heterogeneous treatment effect analysis across different types of housing occupancy 41

Supplementary Note 13. Appendix 44

References 44

Supplementary Note 1. Brief Introduction to the Demand Response

With the reform of the electricity market, the demand response (DR) continues to draw people's attention. The power system must maintain a balance between supply and demand at all times. China and other countries are now faced with the dual pressures of the underutilization of new installed capacity on the power grid supply side and increasing energy consumption on the demand side, creating challenges in matching supply with demand. The traditional approach is to increase the output of the generator set when the load demand is high, but the peak load tends to last for a short time. Increasing energy investment is costly, and the installation of new power plants will increase carbon emissions. Therefore, power departments have begun to explore DR measures to reduce or delay the peak load on the demand side to achieve a balance between supply and demand. Specifically, DR measures can be divided into two categories: price-based DR measures and incentive-based DR measures.

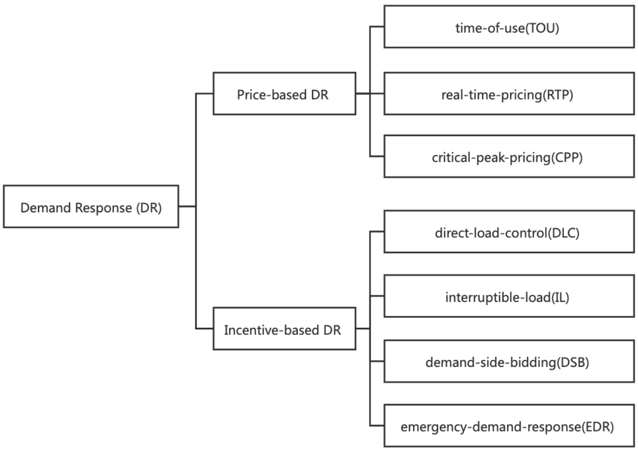

Supplementary Fig. 1 DR categories.

(1) Price-based Demand Response

- TOU (time of use): This daily energy or energy and demand rates are differentiated by peak and off-peak (and possibly shoulder) periods.
- RTP (real time pricing): RTP links hourly prices to hourly changes in the day-of (real-time) or day-ahead cost of power.
- CPP (critical peak pricing): CPP is an overlay on either TOU or flat pricing. CPP uses real-time prices at times of extreme system peak. CPP is restricted to a small number of hours per year, is much higher than a normal peak price, and its timing is unknown ahead of being called.

(2) Incentive-based Demand Response

- DLC (direct load control): Remote and direct control of user's electrical equipment to avoid electricity peaks.
- IL (interruptible load): Similar to DLC, but requires user consent to control the device.
- DSB (demand side bidding): Change the electricity consumption mode and actively participate in market competition in the form of bidding.
- EDR (emergency demand response): When the stability of the power system is threatened, the power department provides compensation for users to reduce the load, and users can choose to participate or give up voluntarily.

At present, the implementation of DR measures is restricted by the power markets of various countries. In countries and regions where the electricity market is regulated,

real-time pricing (RTP), critical peak pricing (CPP), demand-side bidding (DSB), etc. are relatively less popular than TOU; direct load control (DLC) is widely used in the industrial fields of various countries. Industrial users can quickly respond to price or incentive signals and reduce a large amount of their electricity demand. The most commonly used DLC projects involve the remote control and adjustment of electrical equipment such as air conditioners, water heaters, and pool pumps. The EDR projects have some applications in data centers and industrial enterprises, but there are relatively few large-scale experiments on residential users.

From cost-benefit point of view, incentive-based EDR on the residential side is an effective supplement to the electricity price policy in a restricted electricity market like China. The program helps slow down the need to build new power plants to meet short-term spikes in summer and reduce the electricity consumption loss proportion from industrial sector. Compare to power plant construction cost and corporate profits and tax, the rebate cost of the EDR is minimal. Besides, EDR only pays a rebate for households who choose to participate, and may thus be a useful input for informed decision-making balancing the costs and benefits of peak load reduction.

Supplementary Fig. 2 shows the collaboration of various participants in the DR system.

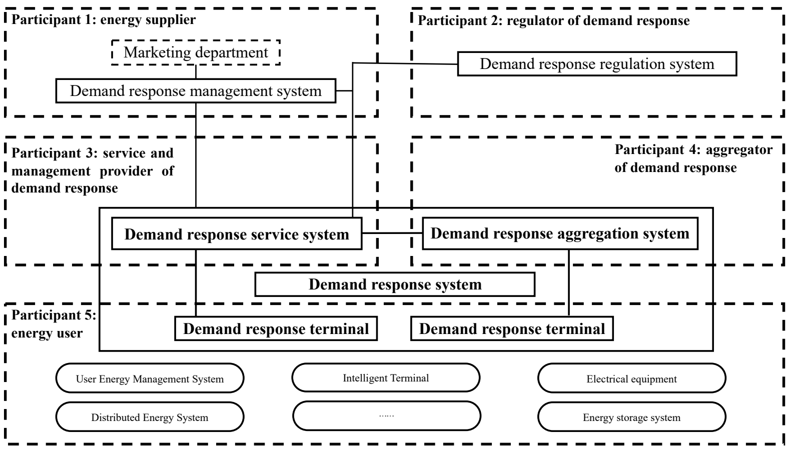

Supplementary Fig. 2 Participants in the DR system.

Energy suppliers use the DR management system to release information such as the DR policy, price and load demand to the DR service system to monitor the DR implementation effect. Regulators play the role of guiding and supervising the DR process conducted by energy suppliers and service and management providers. The service and management providers release DR event information such as dynamic electricity prices and load demands through the DR service system to monitor the operation status and to evaluate performance. The DR aggregation system centralizes the distributed response resources on the demand side and organizes the resources to respond based on the DR event information. Energy users participate in the DR through the DR terminal, which can receive the event information sent by the DR service system or the DR aggregation system. This subsystem interacts with electrical equipment, intelligent terminals and user energy management systems.

Supplementary Note 2. Experiment Description

Note 2.1 Procedure

We carried out this research with the state grid. We first adopted the clustered randomization method to randomly select the region of the EDR trial (divided by communities, a total of 205,129 households were selected) and then installed HPLC smart meters, which can collect electricity consumption information at 15-minute intervals. We randomly assigned permission to apply for the EDR trial and got three samples (see Supplementary Note 2.2), where sample C did not receive any EDR messages during the entire trial period.

Before the experimental period began, we informed households who won the random assignment permission of how they were going to receive the treatments. First, their treatment hours were predetermined—8 pm to 9.30 pm for the summer, corresponding to the system peak-demand hours in the pilot areas (Supplementary Fig. 8B). Second, we defined the treatment days as follows. A treatment day had to be a weekday on which the day-ahead maximum temperature forecast exceeded 35°C (95°F) for the summer.

To understand when and how households received the notifications of their treatments, consider an example treatment date, August 19. On the day before (August 18), the forecasted maximum temperature for August 19 was reported to be above the cutoff level for treatment days. We delivered notifications to households from 8 am to 12 am on August 19 by a text message to their cell phones. Of these households, those that replied "yes" to confirm their participation were considered to be treated. Those that did not reply or that replied with an irrelevant message were not considered to be treated. The English translation of the recruitment message is as follows: "*Dear [name] (user_id), the state grid sincerely invites you to participate in the emergency demand response and monetary reward activity from 20:00 to 21:30 on August 19th. If you use less electricity during this period than during the same period on the 18th, you will receive a monetary reward (reward money is $0.143/kWh), and the money will return to your electricity bill account after the activity ends. Please reply "yes" to confirm your participation in the activity (response valid before 18:00 on August 19*)".

After the trial ended, we distributed rewards that could be used to directly lower the electricity bill in the next month to the household's electronic account. In China, every household has a virtual electricity account (not an electronic bank account), which is used by households to query and pay their electricity bills. The virtual electricity account is owned by every household; thus, there is no issue of the distribution of unbanked users potentially contributing to the observed differences between urban and rural households. In addition, all households must pay their own electricity bills. There may exist two situations: (a) If a household resides in its own house, the owner of the house must pay the household's own electricity bill. (b) In the other situation, the house is rented out. In the area where we were conducting the trial, based on the requirements of leasing companies, tenants must bear the costs of water, electricity and heating during the renting period in addition to rent.

There was no withdrawal mechanism in our trial, which would have limited the effect on the estimation results. First, (a) EDR provides households extra monetary; even if they did not respond or left the study, it would not cause financial losses. Second, (b) the period of the EDR was very short. Households received information on the morning of the treatment day, and that night was the declared treatment period. Most households would not change their choices within such a short period.

We used the exact same procedures to conduct six EDR trials based on monetary rewards in southwestern China from July 18, 2019, to August 21, 2019. The number of invitations was expanded on the basis of the last event (that is, we continued to invite households that were invited last time). The date, daily maximum temperature and participation rate of each trial are as follows:

Supplementary Table 1. Overview of the emergency demand response trials.

Time	Benchmark day		Treatment day		EDR trial		
	Date	Daily maximum temperature (°C)	Date	Daily maximum temperature (°C)	Invitations	Reply	The rate of participation
1	18/7/2019	38.1	19/7/2019	37.9	805	102	12.7%
2	25/7/2019	37.4	26/7/2019	38.7	2577	423	16.4%
3	31/7/2019	38.2	1/8/2019	37.6	3796	596	15.7%
4	31/7/2019	38.2	2/8/2019	37.8	3796	691	18.2%
5	7/8/2019	38.9	8/8/2019	38.4	21703	3820	17.6%
6	18/8/2019	39.6	19/8/2019	39.0	147521	24931	16.9%

Notes. The rate of participation = replies/invitations × 100%; we conducted six EDR trials. The rate of participation was approximately 15%. During the trials, there were persistent high temperatures in the area, with daily maximum temperatures above 35°C.

Survey. A survey investigating the demographic characteristics of the households and assessing their electricity consumption habits was administered between July and December 2019 (see Supplementary Note 11). We conducted this survey (10,254 questionnaires were distributed) mainly through online forms, and the scope of the survey was randomly selected households in the EDR pilot areas. After the surveys were collected (8,548 questionnaires were returned), we tested the validity of the surveys through a rigorous screening process. For surveys with lower validity, we returned them to the participants to complete them again to ensure the quality and quantity of the survey samples. Finally, we obtained 7,774 valid questionnaires.

The rigorous screening process included the following steps: (1) The household had to fill in the correct meter number so that we could match the questionnaire with the electricity use data. (2) The answer time had to be more than 60 seconds. (3) No more than 10% of the questionnaire could be incomplete. (4) Questionnaires were excluded if the family member structure was unreasonable; for example, all family members were under the age of 18. (5) Questionnaires were excluded if there were inconsistent answers to trap items in the questionnaire. For example, in a previous item, the respondent answered that he/she used an electric water heater for showering, but the number of electric water heaters indicated in a following item was zero.

Ethics statement. The State Grid Institutional Review Board approved the experiments described in this article (including all trial households and survey). Informed consent was obtained from all the trial respondents when we started the trial.

Note 2.2 Sample Description

The EDR program was designed as a randomized control trial featuring three sample groups (each of which served a different purpose, as discussed below). We preprocessed the electricity use data (vacant homes that were always at 0 kWh were removed) and deleted the households with missing values caused by collection and transmission by HPLC smart meters.

Sample A (EDR group): The 16,072 households in this group installed an advanced HPLC meter and received the "EDR: economic incentives for energy conservation" message. Those in this group confirmed their participation.

Sample B (no-reply group): The 93,852 households in this group installed an advanced HPLC meter and received an "EDR: economic incentives for energy conservation" message. Those in this group did not confirm their participation.

Sample C (no-notification group): The 95,205 households in this group installed an advanced HPLC meter. Those in this group received no other treatment.

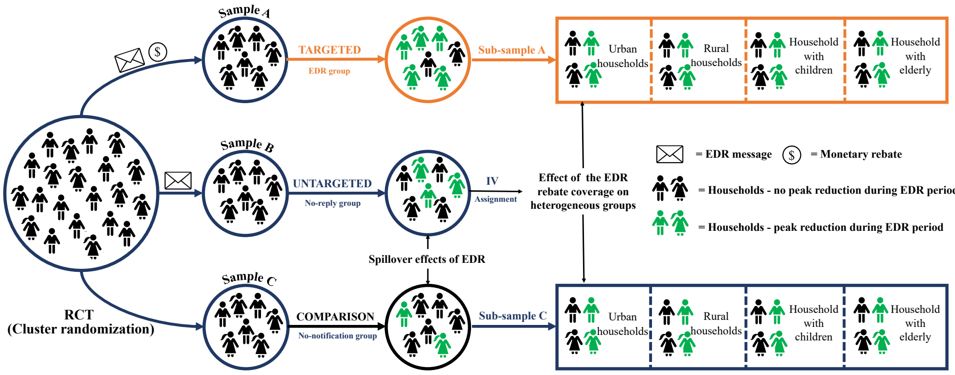

Supplementary Fig. 3 Overview illustration of the assignment process and sample distribution. Sample A (EDR group): The households in this group installed an advanced HPLC meter and received an "EDR: economic incentives for energy conservation" message. Those in this group confirmed their participation. **Sample B (no-reply group)**: The households in this group installed an advanced HPLC meter and received an "EDR: economic incentives for energy conservation" message. Those in this group did not confirm their participation. **Sample C (no-notification group)**: The households in this group installed an advanced HPLC meter. Those in this group received no other treatment. Samples A and B are divided into four types of heterogeneous subsamples: urban households, rural households, households with children, and households with elderly individuals.

The households in sample B were aware of the EDR message but did not reply to us. For this reason, we refer to this group as the no-reply group. In addition, the households in sample C were not made aware of the trial. For this reason, we refer to this group as the no-notification group.

Samples A and B are divided into four subsamples based on the household type (urban households, rural households, households with children, and households with elderly individuals). Based on China's household registration system, households are divided into rural households and urban households based on the *state administrative system*. The households in sample A received a reward of $0.143/kWh for measured electricity reductions during the declared on-peak times. Electricity reductions for reward determination purposes were calculated using the household baseline methodology as follows: The baseline level of usage for each household was calculated based on the household's electricity consumption between 8 pm and 9.30 pm the day before the EDR event.

Supplementary Note 3. Global Final Electricity Consumption and the Temporal Load in the EU and Some Neighboring Countries

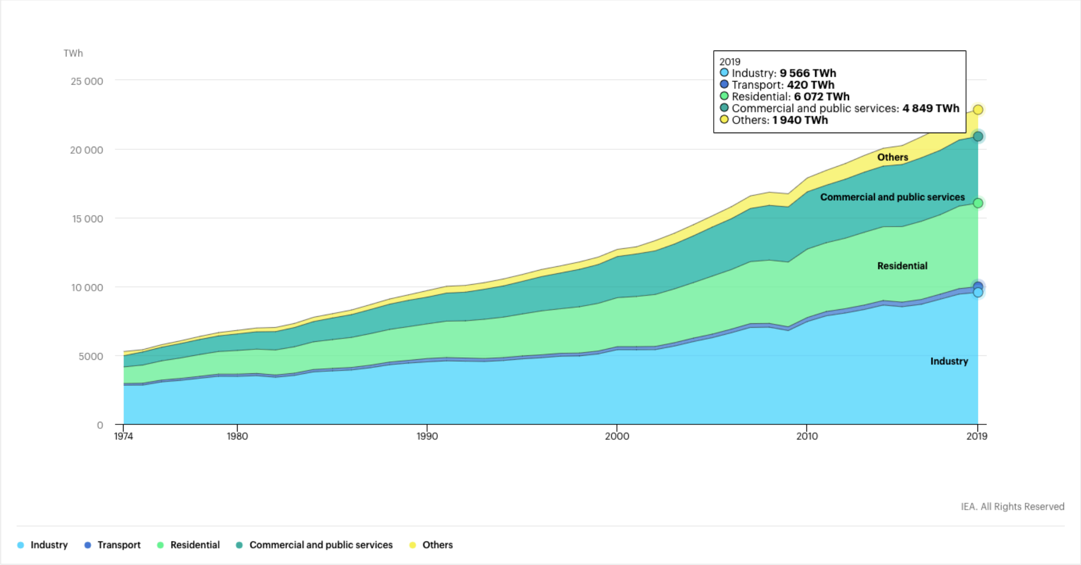

Supplementary Fig. 4 Global final electricity consumption by sector. Source: IEA, World electricity final consumption by sector, 1974-2019, IEA, Paris https://www.iea.org/data-and-statistics/charts/world-electricity-final-consumption-by-sector-1974-2019.

Data on electricity consumption—the monthly aggregated hourly load by country—originate from the *European Network of Transmission Systems Operators for Electricity* (https://www.entsoe.eu/data/power-stats/).

The database covers the 2006-2018 period for 36 countries, while some countries have data only for incomplete years. Within the 2016-2018 period, data points are occasionally missing for several hours or days. If the amount of missing data was 5% or more, we excluded the country from the analysis to avoid distortions. Albania had only one data point in 2016; thus, the on-peak hours of Albania in 2016 were excluded from the analysis because of data scarcity. A comprehensive list of all countries and their respective load data coverage is given in Supplementary Table 2. On-peak hours were calculated as follows: Hourly electricity consumption is more than the country's average annual electricity consumption plus 2 times the standard deviation of annual electricity consumption.

Supplementary Table 2. List of countries and temporal load data coverage.

Country name	ISO code	Entso-e data coverage	Hours in coverage period	(on-peak hours, %) in 2016	(on-peak hours, %) in 2017	(on-peak hours, %) in 2018
Albania	ALB	2016-2018	17519	/	2.76%	2.12%
Austria	AUT	2016-2018	26304	1.08%	2.07%	2.53%
Bosnia Herzeg.	BIH	2016-2018	26304	1.92%	2.00%	0.86%
Belgium	BEL	2016-2018	26304	1.00%	2.35%	3.01%
Bulgaria	BGR	2016-2018	26304	4.80%	5.09%	6.13%
Switzerland	CHE	2016-2018	26304	3.38%	3.13%	4.01%
Cyprus	CYP	2016-2018	25268	3.97%	3.94%	1.93%
Czech Republic	CZE	2016-2018	26304	2.07%	2.71%	2.28%
Germany	DEU	2016-2018	26304	0.05%	0.02%	0.10%
Denmark	DNK	2016-2018	26304	1.55%	1.47%	2.23%
Estonia	EST	2016-2018	26301	2.42%	1.39%	2.31%
Spain	ESP	2016-2018	26304	0.58%	1.56%	1.61%
Finland	FIN	2016-2018	26304	3.40%	1.59%	4.28%
France	FRA	2016-2018	26304	2.22%	3.88%	5.34%
United Kingdom	GBR	2016-2018	26304	2.78%	3.40%	4.29%
Greece	GRC	2016-2018	26302	3.04%	3.94%	1.66%
Croatia	HRV	2016-2018	26304	1.45%	2.13%	2.03%
Hungary	HUN	2016-2018	26304	0.54%	0.61%	1.18%
Ireland	IRL	2016-2018	26304	1.80%	1.71%	1.74%
Iceland	ISL	2016-2018	26304	1.64%	3.00%	3.24%
Italy	ITA	2016-2018	26304	0.58%	0.90%	0.70%
Lithuania	LTU	2016-2018	26304	0.79%	0.43%	1.40%
Luxembourg	LUX	2016-2018	26303	0.90%	1.84%	1.66%
Latvia	LVA	2016-2018	26304	1.49%	0.70%	1.07%
Montenegro	MNE	2016-2018	26304	1.92%	3.13%	2.41%
Macedonia	MKD	2016-2018	26304	4.09%	3.89%	7.45%
Netherlands	NLD	2016-2018	26304	0.96%	1.93%	3.57%
Norway	NOR	2016-2018	26304	2.86%	2.75%	1.32%
Poland	POL	2016-2018	26304	0.43%	0.31%	0.25%
Portugal	PRT	2016-2018	26304	1.14%	2.10%	2.51%
Romania	ROM	2016-2018	26304	2.61%	2.84%	3.93%
Serbia	SRB	2016-2018	26304	2.41%	3.09%	1.39%
Sweden	SWE	2016-2018	26303	2.77%	1.87%	1.71%
Slovenia	SVN	2016-2018	26304	0.30%	1.12%	1.35%
Slovakia	SVK	2016-2018	26304	2.38%	2.34%	3.03%
Turkey	TUR	2016-2018	26304	2.04%	2.12%	2.41%

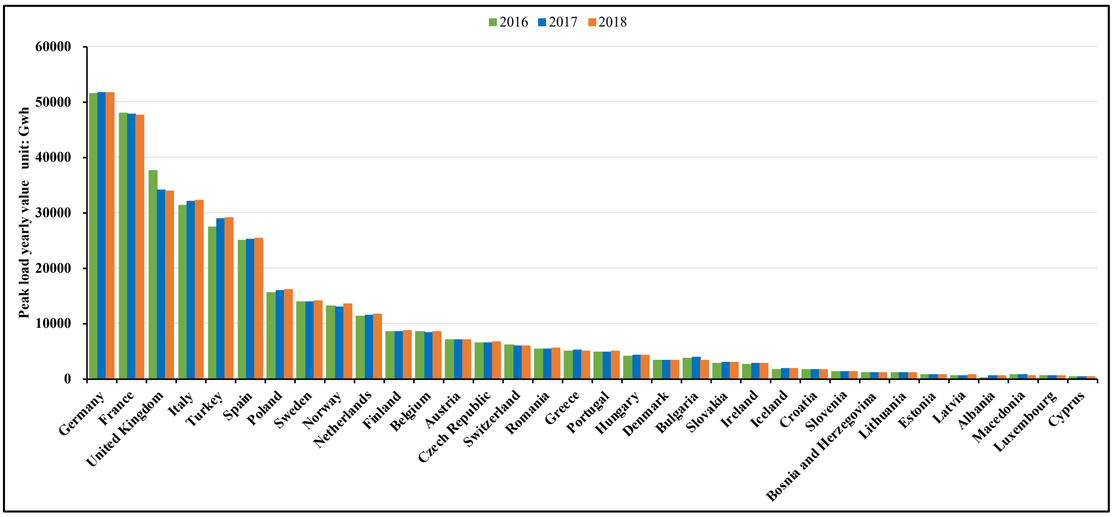

Supplementary Fig. 5 Yearly load of the EU and some neighboring countries (2016-2018). Source: European Network of Transmission Systems Operators.

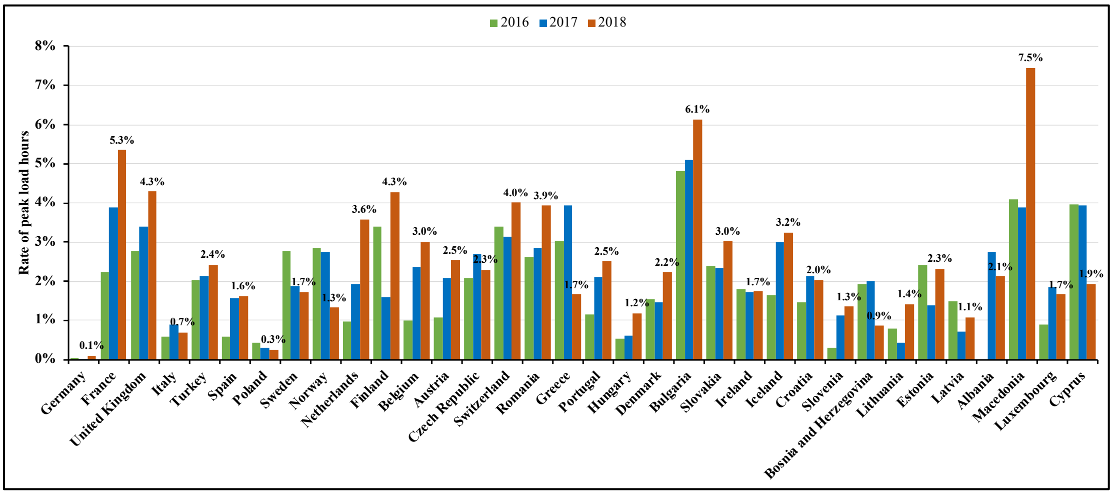

Supplementary Fig. 6 Ratio of the on-peak hours of the EU and some neighboring countries (2016-2018). Source: European Network of Transmission Systems Operators.

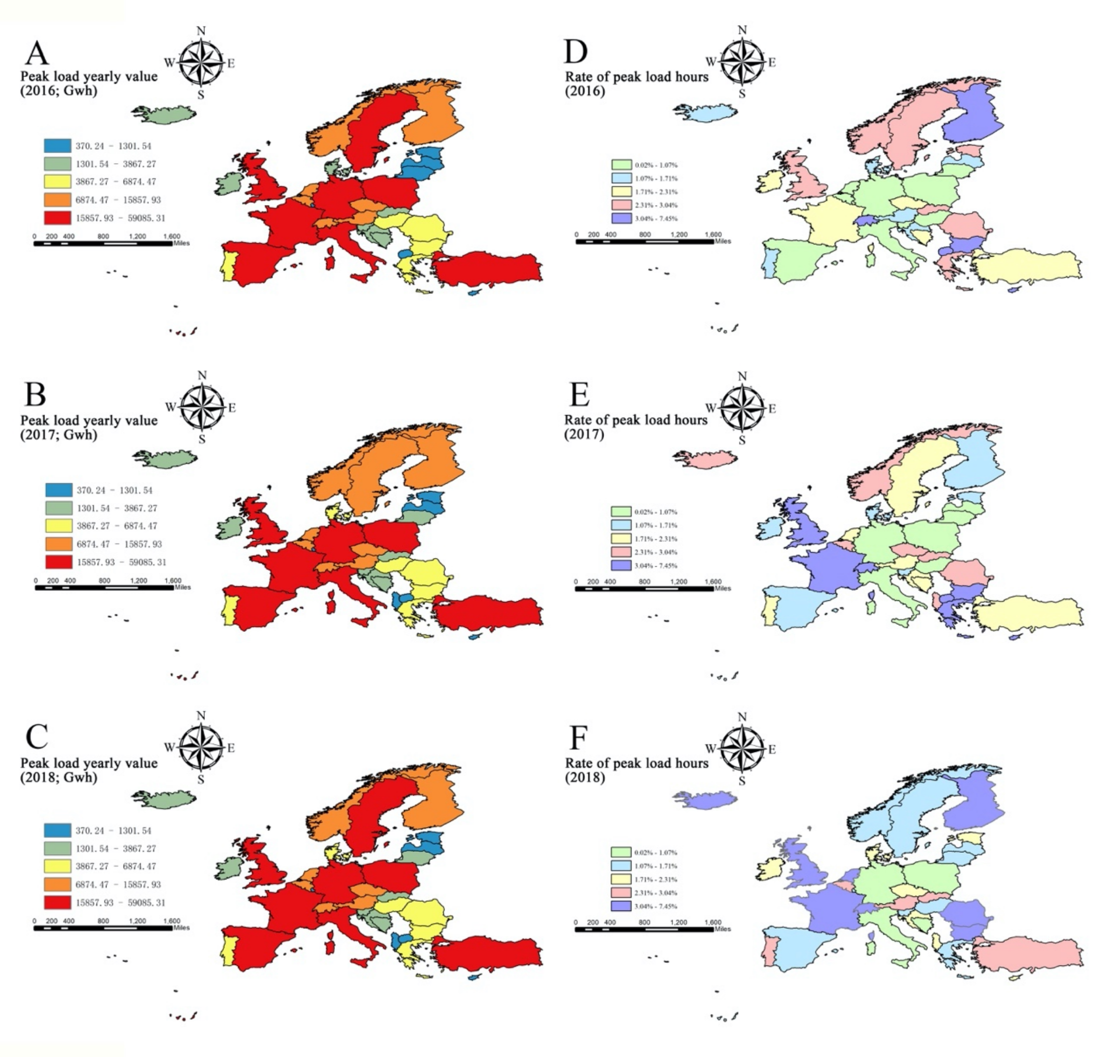

Supplementary Fig. 7 Yearly load and ratio of the on-peak hours of the EU and some neighboring countries (2016-2018). A, B, and C show the yearly load of each country. Some countries exceed 15,857 GWh, as shown in red. **D, E, and F** show the ratio of on-peak hours of each country. Only a few countries are above 3.04%, as shown in purple.

Supplementary Note 4. Relationship between Temperature and Electricity Use

Note 4.1 Estimates of households' electricity use during hot spells

Extreme weather is the major challenge in balancing power supply and demand[1]. Among the categories of reasons, incremental cooling consumption in the residential sector is often recognized as a top component of peak load[2,3,4,5]. Several existing studies have provided empirical evidence on electricity-temperature response functions in advanced economies[6,7], mainly the United States[8,9] and European countries[10,11]. In contrast, we know little about the response functions in China, especially in on-peak times during hot spells. In our experiment in southwestern China, the daily maximum power load increased, while the daily maximum temperature increased, and the daily maximum temperatures of 39°C contributed 21.3 GWh to the daily maximum power loads (Supplementary Fig. 8A). The peak load usually appears between 8 pm and 9.30 pm, which are called on-peak times (Supplementary Fig. 8B). In order to reduce peak load and explore the energy-saving potential of households during on-peak times, we set the period for emergency demand response trial to 8 pm - 9.30 pm. We obtained data from the state grid in the pilot areas and meteorological monitoring stations. The dates are from July 18, 2019, to August 21, 2019.

We collected data on the complete electricity use of 4,097 households (due to the limitation of data availability, the state grid could not provide the data of all households in the pilot areas) in the EDR pilot area for 11 non-EDR days in summer to estimate the relationship between temperature and electricity use. The dates were August 3, 4, 5, 6, 7, 9, 10, 11, 12, 15, and 16, 2019.

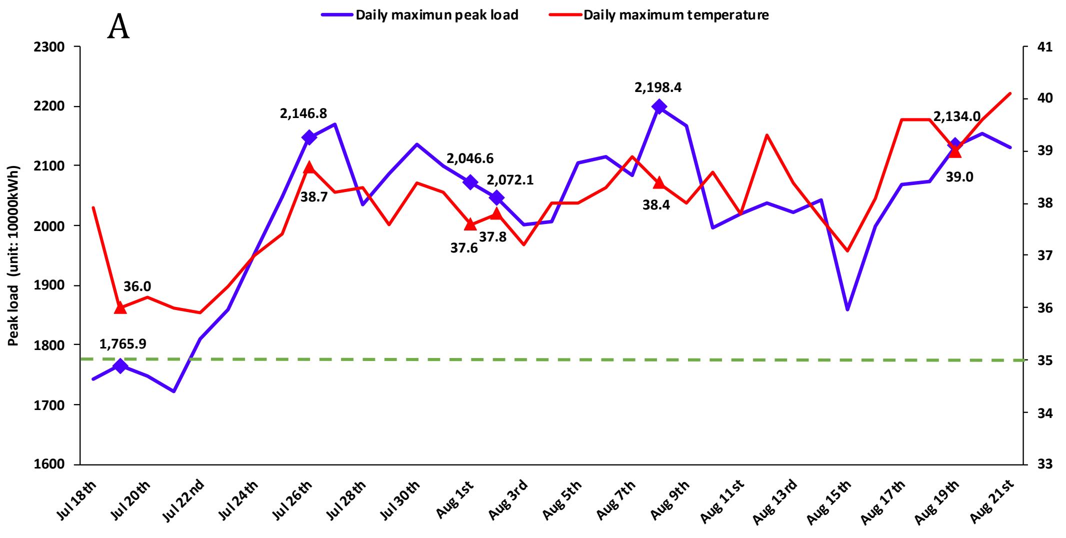

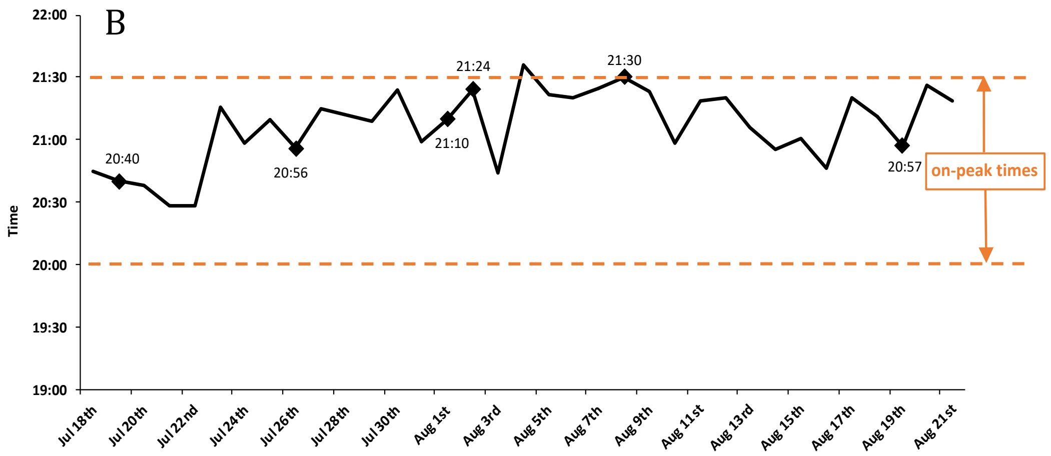

Supplementary Fig. 8 Relationship between temperature changes and electricity use. **A** shows the relationship of the daily maximum temperature and the daily maximum peak power load. The blue solid line indicates the daily maximum peak power load. The red solid line indicates the daily maximum temperature. The green line is the 35°C temperature line. The diamonds and triangles indicate the six EDR trials that we conducted. **B** shows the hours of the total daily maximum power load in the pilot areas. The diamonds indicate the six EDR trials.

The results of Supplementary Table 3 show that households have differences in their electricity use behavioral patterns during on-peak times and off-peak times. Overall, every degree Celsius increase in the ambient temperature in summer leads to approximately 0.665 kWh (0.752 kWh in urban households, 0.404 kWh in rural households) more daily electricity usage compared with the base load in the pilot areas. During on-peak times, every degree Celsius increase in the ambient temperature drives up households' electricity usage in on-peak times by approximately 0.043kWh (0.052kWh in urban households, 0.033kWh in rural households).

Supplementary Table 3. Relationship between temperature and electricity use.

	All times			On-peak times		
	Total	Urban	Rural	Total	Urban	Rural
	(1)	(2)	(3)	(4)	(5)	(6)
Temperature	0.6647***	0.7518***	0.4035***	0.0433***	0.0517***	0.0331***
	(0.0471)	(0.0658)	(0.0634)	(0.0041)	(0.0063)	(0.0052)
Constant	66.4079	91.6097	25.4740	-1.2448	-1.6696	2.9685
	(16.4579)	(26.2488)	(19.8484)	(1.6304)	(2.9798)	(1.8750)
Household FE	Yes	Yes	Yes	Yes	Yes	Yes
Controls	Yes	Yes	Yes	Yes	Yes	Yes
Observations	45,036	25,983	19,053	44,288	26,004	18,284
R-squared	0.0803	0.0399	0.0379	0.0066	0.0049	0.0053

Notes. This table reports the estimated coefficients and cluster-robust standard errors (in parentheses) from Equation (S1) as follows:

$$Elec_{it} = \beta_0 + \beta_1 Temperature_{it} + \beta_2 X_{it} + \alpha_i + \varepsilon_{it} \qquad \text{(Equation (S1))}$$

The coefficients for the *electricity use* of total, urban and rural households at all times and on-peak times (8 pm to 9.30 pm) are presented in columns (1)-(6), respectively. α_i is the individual fixed effect, which refers to those influencing factors that do not change with time, such as age and income. X_{it} represent the remaining control variables, including the air quality and climate related variables, namely, wind direction, wind speed, wind level, relative humidity, atmospheric pressure, vapor pressure, relative humidity and visibility. The term ε_{it} is the error term. The standard errors are clustered at the household level. Significance is at *** p<0.01, ** p<0.05, * p<0.1.

We replace the dependent variable in Equation (S1) with the logarithm of the electricity usage of households, which can approximately interpret the effects in percentage terms. Overall, every degree Celsius increase in the ambient temperature in

summer leads to approximately 5.3% (5.1% in urban households, 3.7% in rural households; Supplementary Table 4) more daily electricity usage compared with the base load in the pilot areas. This result is consistent with the results of previous research[12,13]. Specifically, from 10 pm to 8 am the next day, every degree Celsius increase raises electricity use by more than 5%, and during the remaining hours, it is between 1.6% and 3.2% (Supplementary Table 6).

Supplementary Table 4. Relationship between temperature and the logarithm of electricity use.

	All times			On-peak times		
	Total<br>(1)	Urban<br>(2)	Rural<br>(3)	Total<br>(4)	Urban<br>(5)	Rural<br>(6)
Temperature	0.0528***<br>(0.0055)	0.0510***<br>(0.0071)	0.0367***<br>(0.0083)	0.0213***<br>(0.0055)	0.0200***<br>(0.0077)	0.0279***<br>(0.0085)
Constant	1.7003<br>(2.0789)	2.9008<br>(2.7352)	0.3509<br>(3.2709)	-2.5867<br>(2.3662)	0.5784<br>(3.5245)	-0.3288<br>(3.3274)
Household FE	Yes	Yes	Yes	Yes	Yes	Yes
Controls	Yes	Yes	Yes	Yes	Yes	Yes
Observations	38,478	23,423	15,055	36,922	22,974	13,948
R-squared	0.014	0.012	0.022	0.003	0.003	0.008

Notes: This table reports the estimated coefficients and cluster-robust standard errors (in parentheses) from Equation (S2) as follows:

$$LnElec_{it} = \beta_0 + \beta_1 Temperature_{it} + X_{it} + \alpha_i + \varepsilon_{it} \quad \text{(Equation (S2))}$$

where $LnElec_{it}$ is the electricity use of household i in period t. α_i is the individual fixed effect, which refers to those influencing factors that do not change with time, such as age and income. X_{it} represent the remaining control variables, including climate related variables, namely, wind direction, wind speed, wind level, relative humidity, atmospheric pressure, vapor pressure, relative humidity and visibility. The term ε_{it} is the error term.

The coefficients for logarithm of electricity use of total, urban and rural households in all times and on-peak times (8 pm to 9.30 pm) are presented in columns (1)–(6), respectively. The standard errors are clustered at the household level. Significance is at *** p<0.01, ** p<0.05, * p<0.1.

There are differences in the effect of households' electricity usage by temperature by hour. We drew 24 subgraphs of the relationship between temperature and electricity use by every hour in one day (Supplementary Fig. 9). Hourly electricity use and hourly temperature have a consistent trend correlation. The electricity use from 10 pm to 8 am the next day is more than that in the remaining hours.

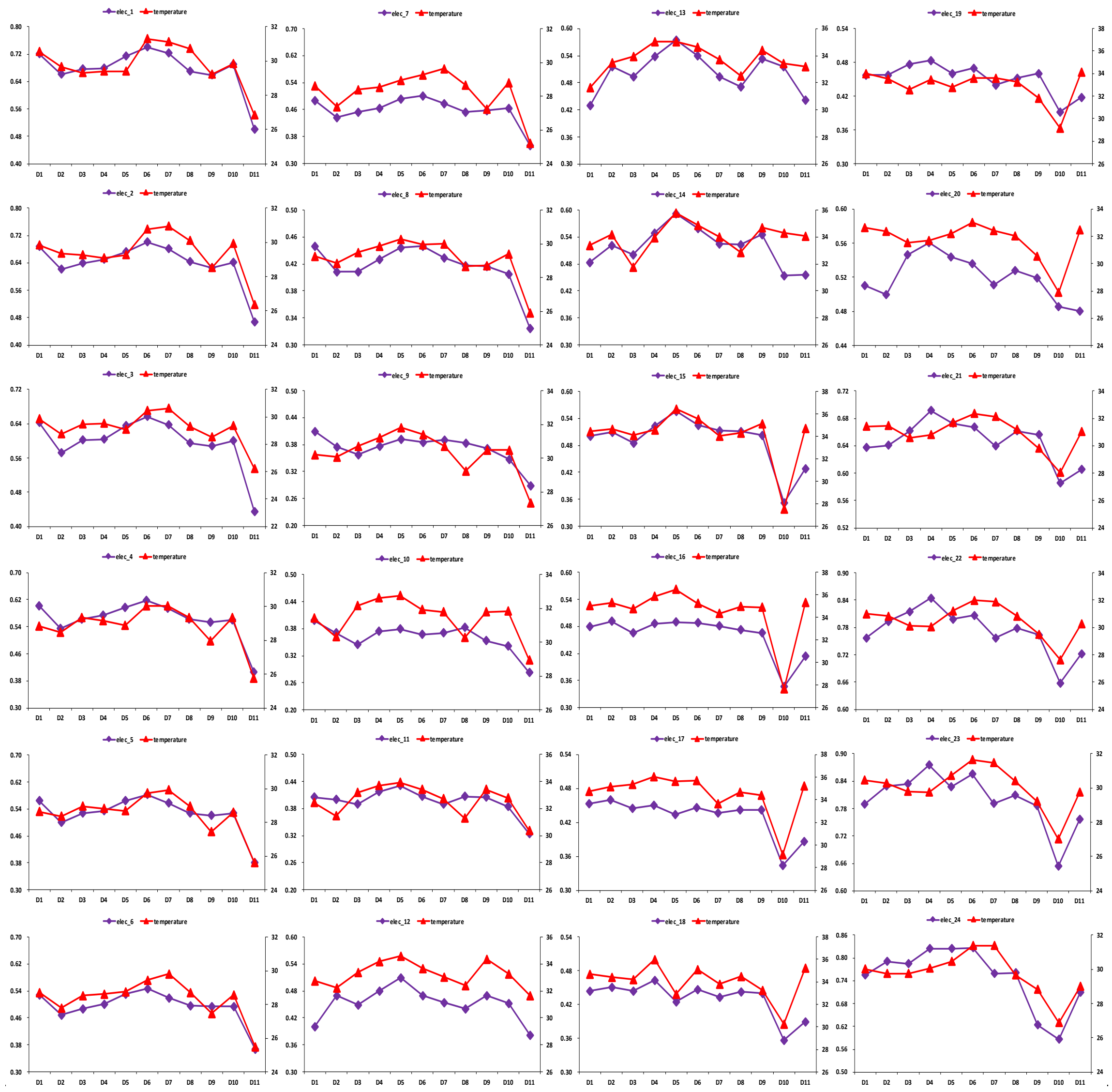

Supplementary Fig. 9 Relationship between temperature and electricity use by hour. The abscissa of each subgraph is the date (11 non-EDR days), the ordinate on the left is the hourly electricity use (unit: kWh), and the ordinate on the right is the hourly temperature (unit: °C).

We built an OLS model to estimate the relationship between temperature and electricity use by hour.

Supplementary Table 5. Relationship between temperature and electricity use by hour.

	Temperature	*SE*	Household FE	Observations	F	R-squared
0am-1am	0.055***	0.0021	Yes	43629	182.50	0.033
1am-2am	0.053***	0.0026	Yes	43352	196.60	0.035
2am-3am	0.046***	0.0021	Yes	43999	189.20	0.036
3am-4am	0.037***	0.0019	Yes	37792	163.10	0.039
4am-5am	0.041***	0.0021	Yes	41779	158.60	0.033
5am-6am	0.034***	0.0019	Yes	41447	141.60	0.029
6am-7am	0.027***	0.0016	Yes	44072	109.50	0.020
7am-8am	0.024***	0.0017	Yes	44717	68.01	0.013
8am-9am	0.016***	0.0022	Yes	44957	44.31	0.009
9am-10am	0.019***	0.0026	Yes	45364	27.98	0.005
10am-11am	0.021***	0.0027	Yes	45364	21.91	0.004
11am-12am	0.035***	0.0031	Yes	45364	33.40	0.006
12am-1pm	0.042***	0.0036	Yes	45363	60.45	0.012
1pm-2pm	0.022***	0.0027	Yes	45363	33.77	0.005
2pm-3pm	0.026***	0.0030	Yes	45364	109.10	0.017
3pm-4pm	0.020***	0.0028	Yes	45337	85.33	0.012
4pm-5pm	0.017***	0.0027	Yes	44691	51.71	0.008
5pm-6pm	0.026***	0.0028	Yes	45364	31.89	0.005
6pm-7pm	0.022***	0.0027	Yes	43905	21.36	0.004
7pm-8pm	0.020***	0.0028	Yes	44584	15.39	0.002
8pm-9pm	0.026***	0.0031	Yes	44958	20.94	0.004
9pm-10pm	0.035***	0.0032	Yes	44645	47.06	0.008
10pm-11pm	0.047***	0.0038	Yes	40415	75.98	0.015
11pm-12pm	0.046***	0.0033	Yes	41851	83.65	0.017

Notes. This table reports the estimated coefficients and cluster-robust standard errors from Equation (S3) as follows:

$$Elec\_H_{it} = \beta_0 + \beta_1 Temperature_{it} + \beta_2 X_{it} + \alpha_i + \varepsilon_{it} \qquad \text{(Equation (S3))}$$

where $Elec\_H_{it}$ is the electricity use of household *i* in hour *t*. α_i is the individual fixed effect, which refers to those influencing factors that do not change with time, such as age and income. X_{it} represent the remaining control variables, including the air quality and climate related variables, namely, wind direction, wind speed, wind level, relative humidity, atmospheric pressure, vapor pressure, relative humidity and visibility. The term ε_{it} is the error term.

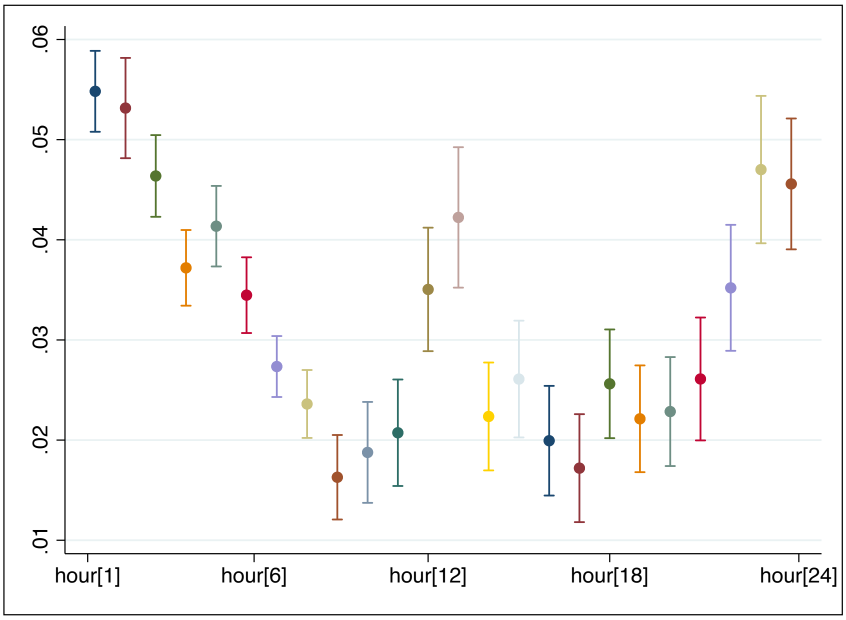

Supplementary Fig. 10 Estimated coefficients of the relationship between temperature and electricity use by hour. The coefficients are for the *electricity use* of households every hour in one day. The standard errors are clustered at the household level. The centers of the error bars are the values of the coefficients, which represent point estimates from the regressions and indicate the average effects of temperature. The vertical lines represent the 95% confidence intervals of the study result, with each end of the line representing the boundaries of the confidence interval. Significance is at *** p<0.01, ** p<0.05, * p<0.1.

We replace the dependent variable in Equation (S3) with the logarithm of electricity usage by hour for households, which can approximately interpret the effects in percentage terms. We built an OLS model to estimate the relationship between temperature and the logarithm of electricity use by hour.

Supplementary Table 6. Relationship between temperature and the logarithm of electricity use by hour.

	Temperature	*SE*	Household FE	Observations	F	R-squared
0am-1am	0.096***	0.0051	Yes	36235	108.80	0.026
1am-2am	0.094***	0.0060	Yes	36095	125.90	0.027
2am-3am	0.098***	0.0054	Yes	36487	126.70	0.029
3am-4am	0.080***	0.0052	Yes	31243	112.30	0.029
4am-5am	0.107***	0.0060	Yes	34523	113.80	0.028
5am-6am	0.077***	0.0052	Yes	34346	96.84	0.022
6am-7am	0.063***	0.0047	Yes	36643	65.74	0.014
7am-8am	0.050***	0.0050	Yes	37152	37.62	0.008
8am-9am	0.021***	0.0057	Yes	37234	31.27	0.008
9am-10am	0.026***	0.0063	Yes	37543	21.44	0.005
10am-11am	0.032***	0.0061	Yes	37550	13.08	0.003
11am-12am	0.042***	0.0069	Yes	37512	19.70	0.004
12am-1pm	0.058***	0.0069	Yes	37382	40.21	0.009
1pm-2pm	0.034***	0.0060	Yes	37458	22.87	0.004
2pm-3pm	0.042***	0.0074	Yes	37483	70.22	0.014
3pm-4pm	0.032***	0.0069	Yes	37478	52.46	0.010
4pm-5pm	0.024***	0.0071	Yes	37102	30.84	0.007
5pm-6pm	0.039***	0.0067	Yes	37539	14.14	0.003
6pm-7pm	0.019***	0.0060	Yes	36407	4.62	0.001
7pm-8pm	0.018***	0.0056	Yes	36307	4.80	0.001
8pm-9pm	0.020***	0.0065	Yes	37133	4.46	0.001
9pm-10pm	0.035***	0.0066	Yes	36941	18.73	0.004
10pm-11pm	0.047***	0.0074	Yes	33560	43.17	0.011
11pm-12pm	0.048***	0.0066	Yes	34679	45.58	0.011

Notes. This table reports the estimated coefficients and cluster-robust standard errors from Equation (S4) as follows:

$$LnElec\_H_{it} = \beta_0 + \beta_1 Temperature_{it} + \beta_2 X_{it} + \alpha_i + \varepsilon_{it} \qquad \text{(Equation (S4))}$$

where $LnElec\_H_{it}$ is the logarithm of electricity use of household i in hour t. α_i is the individual fixed effect, which refers to those influencing factors that do not change with time, such as age and income. X_{it} represent the remaining control variables, including the air quality and climate related variables, namely, wind direction, wind speed, wind level, relative humidity, atmospheric pressure, vapor pressure, relative humidity and visibility. The term ε_{it} is the error term.

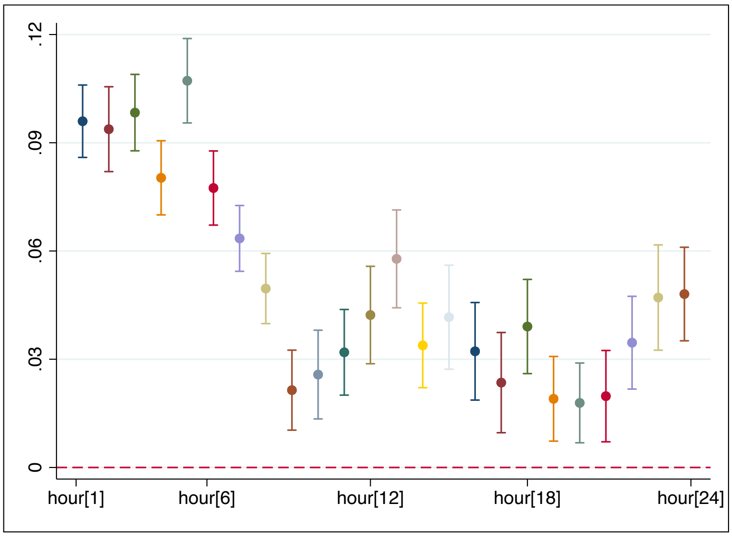

Supplementary Fig. 11 Estimated coefficients of the relationship between temperature and the logarithm of electricity use by hour. The coefficients are for the *logarithm of electricity use* of households every hour in one day. The standard errors are clustered at the household level. The centers of the error bars are the values of the coefficients, which represent point estimates from the regressions and indicate the average effects of temperature. The vertical lines represent the 95% confidence intervals of the study result, with each end of the line representing the boundaries of the confidence interval. Significance is at *** p<0.01, ** p<0.05, * p<0.1.

Note 4.2 Estimate the effect of the EDR when the temperature rises

To estimate the effect of the EDR when the temperature rises, we built the model described by the following equation:

$$Elec_{it} = \beta_0 + \beta_1(Treatment_i \times Post_t \times TempDiff_i) + \beta_2(Treatment_i \times Post_t) + \beta_3(Treatment_i \times TempDiff_i) + \beta_4(Post_t \times TempDiff_i) + \beta_5 Treatment_i + \beta_6 Post_t + \beta_7 TempDiff_i + X_{it} + \varepsilon_{it}$$

(Equation (S5))

where $TempDiff_i$ is a dichotomous variable for the difference in temperature between the treatment day and the benchmark day set to 1 if it is greater than 1 degree Celsius. The term of interest is $TempDiff_i \times Treatment_i \times Post_t$, which indicates the effect of the EDR during on-peak times when the temperature rises. All other variables are as defined in Equation (1) of main text.

Supplementary Table 7. Estimations examining the effects of the EDR on electricity use in areas with temperature rises.

	**EDR assignment selection**		**Heterogeneous effect**			
	**Main effect**	**Spillover effect**	**Urban**	**Rural**	**Children**	**Elderly**
	**(1)**	**(2)**	**(3)**	**(4)**	**(5)**	**(6)**
Treatment ×Post ×TempDiff	0.0196***	0.0054**	0.0103*	-0.0051	0.0396	0.0381
	(0.0035)	(0.0022)	(0.0056)	(0.0081)	(0.0494)	(0.0615)
Treatment ×Post	-0.0192***	-0.0030	-0.0229***	0.0067	-0.0331*	-0.1108***
	(0.0027)	(0.0020)	(0.0028)	(0.0075)	(0.0192)	(0.0230)
TempDiff ×Post	-0.0469**	-0.0313	-0.0974***	0.0430	-0.0897	-0.0095
	(0.0198)	(0.0195)	(0.0273)	(0.0269)	(0.1634)	(0.1618)
TempDiff ×Treatment	0.0064	0.0011	0.0108*	0.0315***	-0.0453	-0.0552
	(0.0040)	(0.0025)	(0.0056)	(0.0088)	(0.0415)	(0.0556)
Treatment	-0.0351***	-0.0244***	-0.0323***	-0.0597***	-0.0175	-0.0141
	(0.0044)	(0.0021)	(0.0046)	(0.0091)	(0.0171)	(0.0183)
Post	-0.0844***	-0.0866***	-0.0783***	-0.1447***	0.0015	-0.0320
	(0.0083)	(0.0091)	(0.0081)	(0.0236)	(0.0484)	(0.0516)
TempDiff	0.1189***	0.1061***	0.1492***	0.0348	-0.0349	-0.0867
	(0.0343)	(0.0361)	(0.0337)	(0.0505)	(0.2230)	(0.1999)
Cluster in group	Yes	Yes	Yes	Yes	Yes	Yes
Controls	Yes	Yes	Yes	Yes	Yes	Yes
Observation	395,072	364,060	299,264	95,808	5,674	5,148
F	49.82	74.66	47.05	32.10	2.474	8.188
R^2	0.0091	0.0086	0.0066	0.0132	0.0108	0.0139

Notes: This table reports the estimated coefficients and cluster-robust standard errors (in parentheses) in Equation (S5). The dependent variable in all columns is electricity usage during on-peak times. Columns (1) and (3-5) estimate the effect of assignment selection comparing the average outcome for households selected in the random assignment (EDR group and no-reply group, which we called assignment winners) to the average outcome for control households (no-notification group those not selected by the assignment) among heterogeneous groups. Column (2) compares the difference between the no-reply group and the no-notification group. The standard errors are clustered at the household-group level. Significance is at *** p<0.01, ** p<0.05, * p<0.1.

We replace the variable $TempDiff_i$ in Equation (S5) with the $Temperature_i$, which can estimate the effect of EDR with increasing temperature. This estimation strategy followed the same logic as a standard difference-in-difference-in-difference (DDD) strategy to examine whether the EDR still worked when the temperature increased. We compared the relative change in electricity conservation of the EDR group in the posttreatment period relative to the pretreatment period when the temperature increased. The difference between our estimates and a standard DDD strategy was that we used a continuous measure of the intensity of the treatment (i.e., the difference in temperature between the treatment day and the benchmark day), thereby capturing more variation in the data.

$$\begin{aligned} Elec_{it} = \beta_0 &+ \beta_1(Treatment_i \times Post_t \times Temperature_i) \\ &+ \beta_2(Treatment_i \times Post_t) + \beta_3(Treatment_i \times Temperature_i) \\ &+ \beta_4(Post_t \times Temperature_i) + \beta_5 Treatment_i + \beta_6 Post_t \\ &+ \beta_7 Temperature_i + X_{it} + \varepsilon_{it} \end{aligned}$$

(Equation (S6))

where $TempDiff_i$ is a continuous variable of the intensity of temperature (i.e., the difference in temperature between the treatment day and the benchmark day). All other variables are as defined in Equation (1) of main text.

Supplementary Table 8. Estimations examining the effects of the EDR on electricity use with increasing temperature.

	EDR assignment selection		Heterogeneous effect			
	**Main effect (1)**	**Spillover effect (2)**	**Urban (3)**	**Rural (4)**	**Children (5)**	**Elderly (6)**
Treatment ×Post ×Temperature	0.0036	0.0071***	0.0019	-0.0010	-0.0455*	-0.0346
	(0.0024)	(0.0022)	(0.0027)	(0.0054)	(0.0271)	(0.0292)
Treatment ×Post	-0.1292*	-0.2267***	-0.0827	0.0429	1.4393*	1.0180
	(0.0768)	(0.0678)	(0.0871)	(0.1704)	(0.8652)	(0.9307)
Temperature ×Post	0.0415***	0.0399***	0.0028	0.1818***	0.0237	-0.0384
	(0.0120)	(0.0123)	(0.0120)	(0.0317)	(0.0792)	(0.0815)
Temperature ×Treatment	-0.0017	-0.0034**	-0.0001	-0.0065*	0.0025	0.0064
	(0.0019)	(0.0015)	(0.0023)	(0.0035)	(0.0216)	(0.0245)
Treatment	0.0211	0.0836*	-0.0280	0.1651	-0.0995	-0.2238
	(0.0577)	(0.0464)	(0.0744)	(0.1066)	(0.6873)	(0.7794)
Post	-1.3542***	-1.3078***	-0.1512	-5.8283***	-0.7887	1.1318
	(0.3776)	(0.3859)	(0.3805)	(1.0146)	(2.5288)	(2.5956)
Temperature	0.1437***	0.1973***	0.1825***	-0.4533***	-0.0901	0.4670
	(0.0500)	(0.0552)	(0.0673)	(0.1274)	(0.3301)	(0.3413)
Cluster in group	Yes	Yes	Yes	Yes	Yes	Yes
Controls	Yes	Yes	Yes	Yes	Yes	Yes
Observation	410,258	378,114	304,000	106,258	6,408	5,978
F	55.60	66.55	51.93	29.27	2.70	8.99
R^2	0.0086	0.0084	0.0065	0.0175	0.0066	0.0095

Notes: This table reports the estimated coefficients and cluster-robust standard errors (in parentheses) in Equation (S6). The dependent variable in all columns is electricity usage during on-peak times. Columns (1) and (3-5) estimate the effect of assignment selection comparing the average outcome for households selected in the random assignment (EDR group and no-reply group, which we called assignment winners) to the average outcome for control households (no-notification group those not selected by the assignment) among heterogeneous groups. Column (2) compares the difference between the no-reply group and the no-notification group. The standard errors are clustered at the household-group level. Significance is at *** p<0.01, ** p<0.05, * p<0.1.

Supplementary Note 5. Descriptive Statistics

Supplementary Table 9. Descriptive statistics of electricity use and weather-related data in the EDR trial.

	Sample in the experiment		Random sample	All (N=205,129; Survey sample N=7,774)
	EDR (N=16,072; Survey subsample N=1,096)	no-reply group (N=93,852; Survey subsample N=3,578)	no-notification group (N=95,205; Survey subsample N=3,099)	
Frequencies (%)				Range
Rural households	16%	27%	26%	26%
Urban households	84%	73%	74%	74%
Households with elderly (Survey)	36%491	35%1292	39%1208	38%
Households with children (Survey)	39%432	40%1438	43%1332	41%
Mean (SD) benchmark day				
Monthly electricity use	214.67(147.34)	200.26(136.64)	199.45(135.07)	1 to 1000 kWh
Temperature	31.58(0.89)	31.51(0.94)	31.55(0.96)	30.2 to 33.2 ℃
Electricity use (kWh/on-peak times)	1.12(1.19)	1.11(1.16)	1.13(1.16)	0 to 10.24 kWh
Electricity use (Δ< 1 ℃)	1.16(1.22)	1.18(1.22)	1.19(1.21)	0 to 9.97 kWh
Electricity use (Δ≥ 1 ℃)	0.98(1.00)	0.97(0.97)	0.99(1.00)	0 to 10.24 kWh
Mean (SD) treatment day				
Temperature	31.88(0.96)	32.00(0.92)	31.97(0.92)	29.9 to 33.4 ℃
Electricity use (kWh/on-peak times)	0.99(1.16)	1.07(1.15)	1.09(1.14)	0 to 12.29 kWh
Electricity use (Δ< 1 ℃)	1.00(1.18)	1.10(1.20)	1.15(1.20)	0 to 10.14 kWh
Electricity use (Δ≥ 1 ℃)	0.93(0.98)	1.00(0.97)	0.96(0.97)	0 to 12.29 kWh
Total load (kWh/ on-peak times)				
Load (benchmark day)	18068.48	104352.00	107419.00	229839.50
Load (treatment day)	15983.84	100884.70	104071.00	220939.60
% load reduction	11.54%	3.32%	3.12%	3.87%

Notes. Columns (1) and (2) show the sample mean and standard deviation of the observables for the EDR group and no-reply group. On the benchmark day, the observables are balanced across the groups. Column (3) shows the mean and standard deviation of the observables for the no-notification group (a random sample in the experimental areas). Column (4) presents the mean and standard deviation of the observables for all samples. Standard deviations are in parentheses.

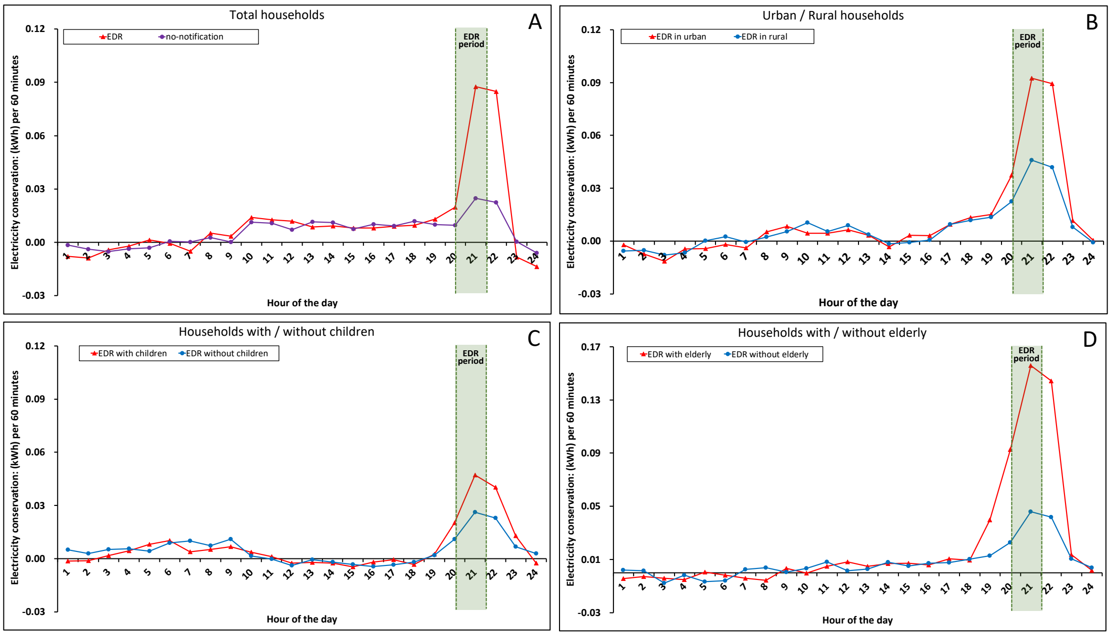

Supplementary Fig. 12 Effect of the incentive-based EDR on electricity usage among heterogeneous groups. A-D show the electricity conservation of the EDR group and no-notification group among heterogeneous groups. The ordinate is the difference in electricity usage (kWh) for 60-minute intervals between the benchmark day and the treatment day, which means a positive value refers to a reduction in electricity use. **A** represents the overall effect. The red line represents the EDR group, and the purple line represents the no-notification group. **B, C, and D** represent the heterogeneous effects on urban households, households with children, households with elderly individuals and the control group, respectively.

Supplementary Note 6. Methods and Endogeneity Problems

Our main regression specifications leverage the random assignment to make comparisons between the treatment and control group. The random assignment supported permission to apply for the EDR rebate program. We can estimate **the effect of random assignment selection** by fitting difference-in-difference regressions and comparing the average outcome for all households selected in the random assignment (EDR group and no-reply group in our trial, which we called assignment winners, Supplementary Fig. 3) to the average outcome for all control households (no-notification, those not selected by the assignment). This is an intent-to-treat estimate (results in Table 1 and Supplementary Tables 12-13). We can estimate the effect of EDR rebate coverage by fitting two-stage least squares regressions (with random assignment selection as an instrument for EDR rebate coverage) and estimating **the local average treatment effect of EDR rebate coverage** (results in Table 2 and Supplementary Tables 10-11, 14). Both approaches use the randomization of the assignment to estimate causal effects.

Note 6.1 Analytic specifications: Effect of random assignment selection (intent to treat)

We estimate **the intent-to-treat effect of random assignment selection** (i.e. the difference between treatment and controls) by fitting the following difference-in-difference equation:

$$Elec_{it} = \beta_0 + \beta_1(Treatment_i \times Post_t) + \beta_2 Treatment_i + \beta_3 Post_t + X_{it} + \varepsilon_{it} \quad \text{(Equation (S7))}$$

where i denotes a household. $Treatment_i$ is a dichotomous variable set to 1 if the household was selected by the random assignment and 0 if the household was in the control group. The coefficient on $Treatment_i \times Post_t$ (β_1) is the main coefficient of interest, and gives the average difference in means between the treatment group (the assignment winners) and the control group (those not selected by the assignment); it is interpreted as the impact of being able to apply for the EDR program.

Note 6.2 Analytic specifications: Effect of EDR rebate coverage (instrumental variable two-stage approach)

The intent-to-treat estimates from equation (S7) provide an estimate of the causal effect of winning the assignment (i.e., winning permission to apply for the EDR trial). This provides an estimate of the net impact of expanding access to EDR, which may lead to conservative effects of the treatment. We are also interested in the effect of EDR coverage itself. We adopt an instrumental variable two-stage approach to estimate the effect of the treatment - not just assignment to treatment - that must account for noncompliance. To adjust for noncompliance, one can use assignment to treatment as an instrument for treatment receipt since the initial assignment was random. We estimate the effects of EDR by fitting two-stage least squares regressions (with assignment selection as an instrument for EDR rebate coverage) and **estimating the local average treatment effect of EDR rebate coverage**. We model this as follows:

$$Elec_{it} = \pi_0 + \pi_1 Treatment_{it} + X_{it} + \alpha_i + u_{it} \quad \text{(Equation (S8))}$$

where $Treatment$ is defined as participating in the EDR trial during the study period. All other variables are as defined in equation (S7). We estimate equation (S8) by instrumental variable regression using the following first-stage equation:

$$Treatment_{it} = \beta_0 + \beta_1 IV\_assignment_{it} + X_{it} + \alpha_i + v_{it} \text{ (Equation (S9))}$$

in which the excluded instrument is the variable $Assignment$.

We interpret the coefficient on $Treatment_{it}$ from the instrumental variable estimation of equation (S9) as the local average treatment effect of EDR. In other words, our estimate of π_1 identifies the causal effect of EDR rebate coverage among the subset of households who participated in the EDR upon winning the assignment but who would not participate in EDR without winning the assignment (i.e., the compliers).

Note 6.3 Analytic specifications: Other method to solve endogenous problems

When carefully interpreting the size of the treatment effect, we want to emphasize that an important limitation of our experiment is that the subjects were not a random sample of the population (between EDR group and no-notification group), as described in the experimental design section. It's undeniable that there could be unobservable factors that are different between the two groups, while we adopted a DTW-based matching method to control for the parallel trend between the experimental sample and control sample (results in Table 2 and Supplementary Tables 12-14). For example, our sample households could be worried about electricity outages and be potentially more willing to respond to our treatment. If that is the case, the treatment effect on our sample could be larger than that on the population. Similar to previous field experiments in electricity demand, we still need to solve some potential endogeneity problems. We showed that our estimated results are similar to those obtained in previous studies on residential electricity demand. However, this comparison is merely suggestive evidence, and we emphasize that our experiment cannot be entirely free from external validity issues.

1. We adopted a cluster randomization method to evaluate the effect of the EDR trial. EDR trials can be seen as an exogenous shock; however, the selection of the treatment group might not be completely random. For instance, low-income households, households with energy conservation potential, or households that are more sensitive to monetary incentives might be more inclined to participate in the EDR, which might result in self-selection bias. Therefore, we adopted a variety of robustness checks to solve the endogeneity problem of self-selection.
    2.1 Fixed effects. We use household-level fixed effects to control for time-constant and unobserved household characteristics, such as income, occupation, energy preference, and money sensitivity. The results are shown in Supplementary Tables 12-14, and the effect of random assignment (*coef.* = *0.0162*, $p<0.001$, Supplementary Table 12) and the effect of EDR rebate coverage (*coef.* = *0.0940*, $p<0.001$, Supplementary Table 14) are significant and consistent with the conclusion of the paper.
    2.2 Matching. We use matching methods to solve the problem of parallel trends between the treatment group and the control group. It is difficult for traditional

matching methods (such as propensity score matching) to capture all of the factors that affect the electricity use behavior during on-peak times through the covariates. We thus adopt a DTW-based matching method to control for the parallel trend. This method integrates the households' 15-minute high-frequency and monthly low-frequency electricity consumption data on a microscale. From the perspective of behavioral results, we believe that the historical electricity use fluctuation trend contains known, unknown, or difficult-to-measure variables with traditional methods that affect the willingness to participate in the EDR. The households with similar electricity patterns in the long term (36 months) and short term (15 minutes) before the trial should be comparable and conform to the parallel trend. We applied this method to divide all samples into 100 groups of electricity use patterns to ensure that the treatment was randomly distributed within the group.

2.3 Parallel trend test. We use econometric methods to test whether the treatment group and control group conform to the parallel trends. We found that there was no significant difference between the treatment group and the control group before the EDR trial; that is, the two samples conformed to the parallel trend hypothesis (Supplementary Fig. 15; see Supplementary Note 9.1 for details).

2.4 Heckman two-step test. We use the Heckman two-step method as a robustness test to correct for sample self-selection bias (see Supplementary Note 9.2 for details). Heckman's coefficient is significant (*coef. = -0.1239, p = 0.027;* Supplementary Table 16), and the coefficients of other similar methods are also significant (*OLS coefficient. = -0.0844, p = 0.060; HeckMLE coef. = -0.1627, p = 0.033; Heck2SLS coef. = -0.1626, p = 0.033;* Supplementary Table 16). These results are consistent with the conclusion of the paper.

2.5 Placebo test. We perform a placebo test to conduct a counterfactual test by changing the implementation time of the EDR. The results (Supplementary Tables 17-18) show that the key estimated coefficients in each group were not significant, which means that following the removal of the EDR trial, there were no systematic differences in the changes in electricity use between the treatment and control groups. This finding proves that our previous estimation results are robust.

Note. Specific description of the DTW-based matching algorithm

We use DTW-based matching methods to solve the problem of parallel trends between the treatment group and the control group. This process is carried out in two steps. The first step is matching the households' electricity use data and the meteorological data. The second step is using the DTW method based on monthly electricity use data (January 2016 to March 2019) and high-frequency 15-minute electricity use data (160 data points before 8 pm on August 19, 2019), and the households are divided into 100 groups based on the most similar electricity use trends.

We show some results in graphs after clustering. There are some differences in electricity use among the groups, and the households in each group are relatively similar in terms of electricity use trends.

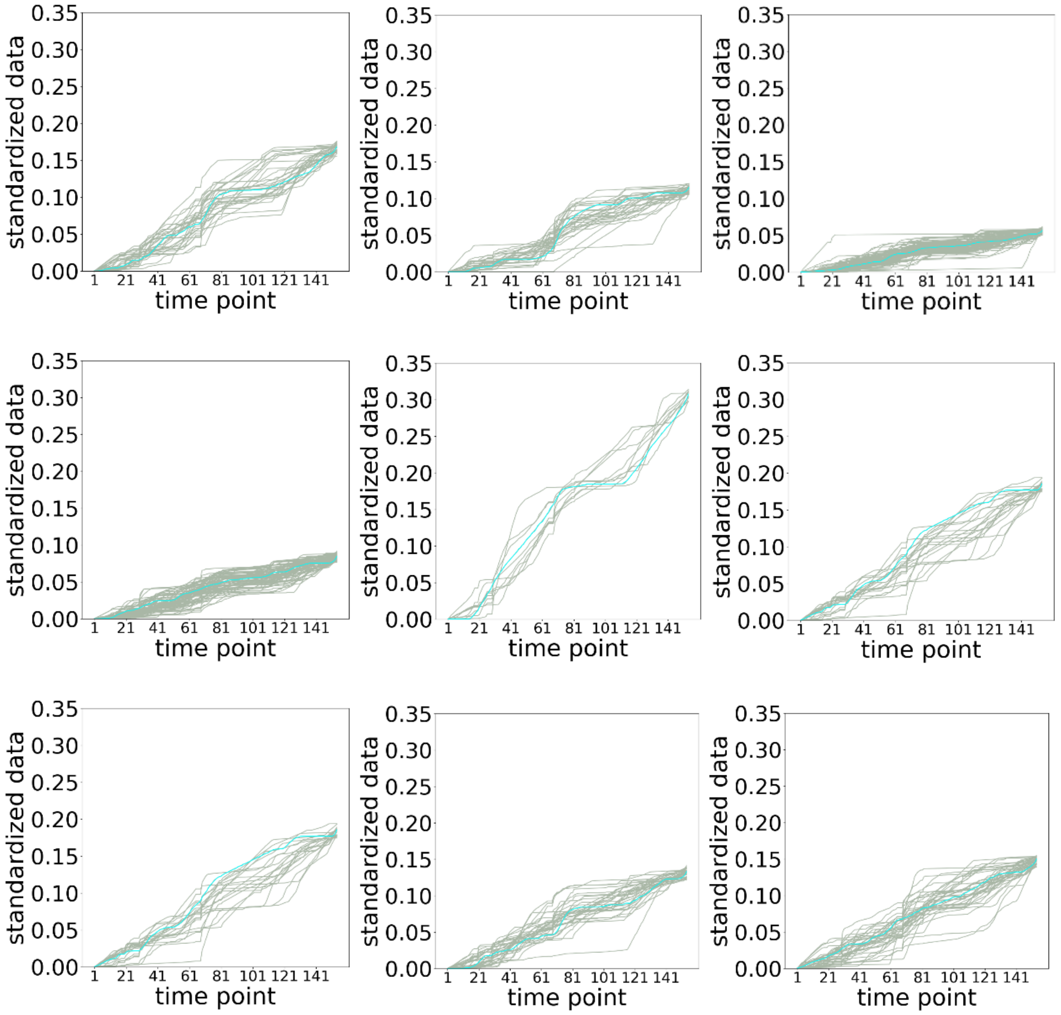

Supplementary Fig. 13 Electricity use behavioral patterns (high frequency) of randomly selected groups of matched households. The cyan line represents the 15-minute interval electricity use curve of the household at the center of the group, and the gray line represents the 15-minute interval electricity use curve of each household.

The clustering results also performed well in monthly electricity use. The monthly electricity use of each group is different, and there is a seasonal distribution characteristic, which shows that DTW-based matching methods can well capture the information on households' electricity use behavior.

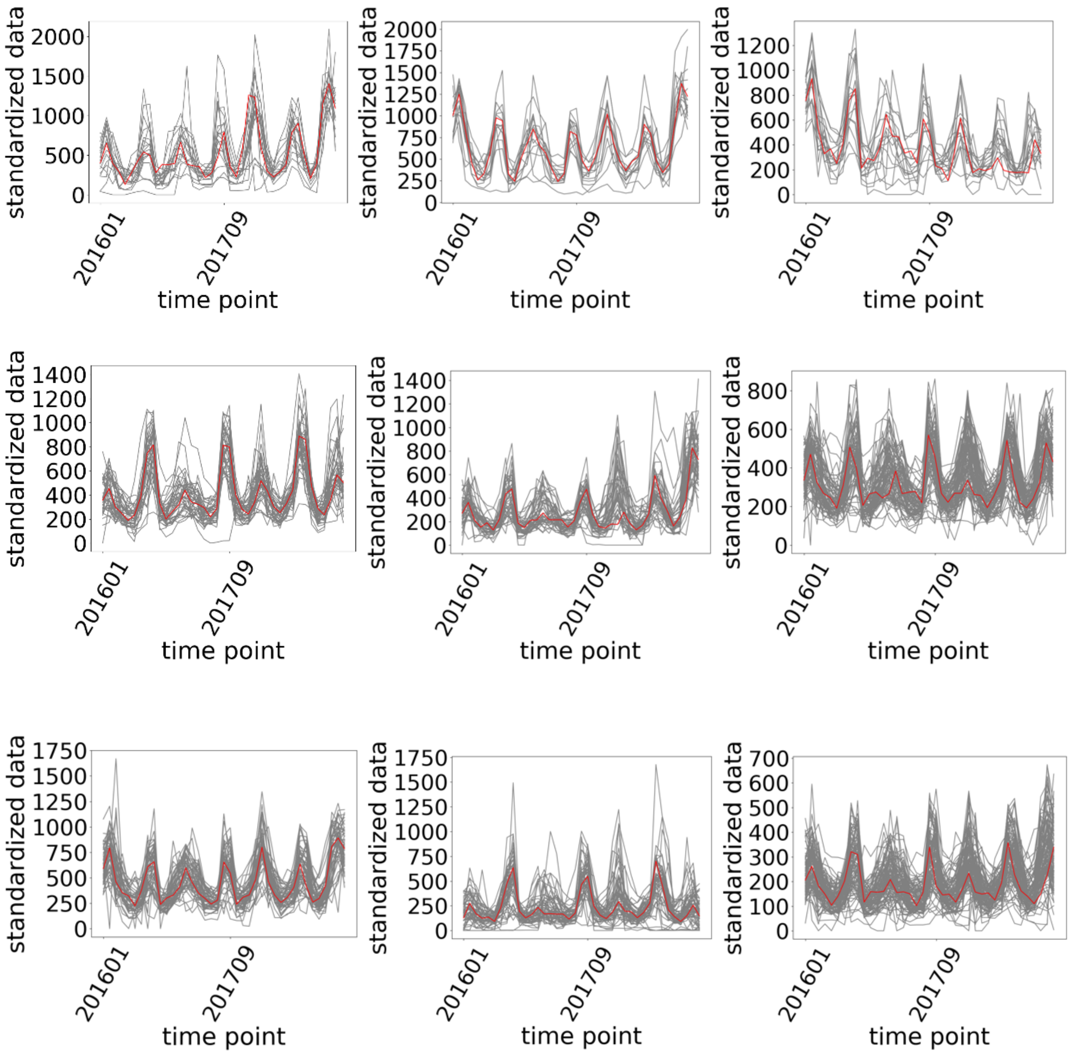

Supplementary Fig. 14 Electricity use behavioral patterns (monthly) of randomly selected groups of matched households. The red line represents the monthly electricity use curve of the household at the center of the group, and the gray line represents the monthly electricity use curve of each household.

Supplementary Note 7. Supplementary Tables of Detailed Results

We replace the dependent variable in Equations (1) and (2) of the main text with the logarithm of electricity usage of households during the declared on-peak times, which can interpret the treatment effects approximately in percentage terms. EDR assignment selection increases electricity use conservation by 1.02 percent relative to the control mean.

Supplementary Table 10. Estimations examining the effects of the EDR assignment selection on the logarithm of electricity use.

	EDR assignment selection		Heterogeneous effect		
	**Main effect**	**Spillover effect**	**Urban**	**Children**	**Elderly**
	(1)	(2)	(3)	(4)	(5)
Treatment ×Post ×Indicator			-0.0090***	-0.0385	-0.0228**
			(0.0013)	(0.0245)	(0.0112)
Treatment ×Post	-0.0102***	-0.0031	-0.0031***	0.0290	-0.0160**
	(0.0011)	(0.0007)	(0.0009)	(0.0235)	(0.0072)
Indicator ×Post			-0.0104**	0.0737**	-0.0219
			(0.0042)	(0.0331)	(0.0164)
Indicator × Treatment			0.0006	0.0134	0.0003
			(0.0017)	(0.0212)	(0.0099)
Treatment	-0.0162***	-0.0122***	-0.0167***	-0.0210	-0.0034
	(0.0022)	(0.0008)	(0.0021)	(0.0200)	(0.0072)
Post	-0.0131***	-0.0138***	-0.0092	-0.0875***	-0.0144
	(0.0045)	(0.0047)	(0.0061)	(0.0324)	(0.0155)
Indicator			-0.0512***	0.0151	0.0702***
			(0.0105)	(0.0339)	(0.0195)
Cluster in group	Yes	Yes	Yes	Yes	Yes
Controls	Yes	Yes	Yes	Yes	Yes
Observation	410,258	378,114	410,258	7,466	10,322
F	90.80	91.90	78.78	3.323	14.77
R^2	0.0095	0.0090	0.0106	0.0098	0.0154

Notes: This table reports the estimated coefficients and cluster-robust standard errors (in parentheses) in Equations (1) and (2). The dependent variable in all columns is the logarithm of electricity usage during declared on-peak times. Columns (1) and (3-5) estimate the effect of assignment selection comparing the average outcome for households selected in the random assignment (EDR group and no-reply group, which we called assignment winners) to the average outcome for control households (no-notification group those not selected by the assignment) among heterogeneous groups. Column (2) compares the difference between the no-reply group and the no-notification group. The standard errors are clustered at the household-group level. Significance is at *** p<0.01, ** p<0.05, * p<0.1.

EDR rebate coverage increases electricity use conservation by 7.32 percent relative to the control mean.

Supplementary Table 11. Estimations examining the effects of the EDR rebate coverage on the logarithm of electricity use.

	EDR rebate coverage (IV two-stage)				EDR rebate coverage (DTW-matching)			
	**Total**	**Urban**	**Children**	**Elderly**	**Total**	**Urban**	**Children**	**Elderly**
	(1)	(2)	(3)	(4)	(5)	(6)	(7)	(8)
Treatment ×Post ×Indicator		-0.0219***	-0.0561	-0.0722**		-0.0266***	-0.0743	-0.0721**
		(0.0083)	(0.0669)	(0.0337)		(0.0080)	(0.0699)	(0.0329)
Treatment ×Post	-0.0732***	-0.0314***	0.0223	-0.0650**	-0.0520***	-0.0272***	0.0403	-0.0654***
	(0.0124)	(0.0075)	(0.0628)	(0.0280)	(0.0060)	(0.0084)	(0.0657)	(0.0242)
Indicator ×Post		0.0100***	0.0603***	-0.0203		-0.0083*	0.0745**	-0.0220
		(0.0023)	(0.0229)	(0.0124)		(0.0042)	(0.0332)	(0.0164)
Indicator ×Treatment						0.0152	-0.0343	-0.0010
						(0.0103)	(0.0671)	(0.0315)
Treatment					-0.0377***	-0.0505***	0.0321	0.0170
					(0.0123)	(0.0171)	(0.0621)	(0.0256)
Post	0.0258***	0.0000	-0.1282**	-0.0477	-0.0123***	-0.0103	-0.0899***	-0.0148
	(0.0092)	(0.0082)	(0.0539)	(0.0440)	(0.0046)	(0.0063)	(0.0324)	(0.0156)
Indicator						-0.0558***	0.0133	0.0698***
						(0.0107)	(0.0347)	(0.0198)
Cluster in group	No	No	No	No	Yes	Yes	Yes	Yes
Controls	Yes	Yes	Yes	Yes	Yes	Yes	Yes	Yes
Observation	410,258	410,258	7,466	10,322	222,554	222,554	4,124	5,814
R^2	0.0048	0.0051	0.0082	0.0266	0.0128	0.0140	0.0088	0.0204

Notes: This table reports the estimated coefficients and cluster-robust standard errors (in parentheses) in Equations (1) and (2). The dependent variable in all columns is the logarithm of electricity usage during declared on-peak times. Columns (1-4) use a standard instrumental-variable approach with assignment selection as an instrument to estimate EDR rebate coverage among heterogeneous

groups. Column (5-8) compares the difference between the EDR group and the no-notification group by using matching methods. The standard errors are clustered at the household-group level. Significance is at *** p<0.01, ** p<0.05, * p<0.1.

EDR rebate coverage increases electricity use conservation by 0.1422 kWh relative to the urban no-notification group. Households with elderly individuals participating in the EDR saved 0.3105 kWh compared to households with elderly individuals in the no-notification group.

Supplementary Table 12. Estimations examining the effects of the EDR rebate coverage on electricity use (within heterogeneous group regression).

	EDR rebate coverage (IV two-stage)				EDR rebate coverage (DTW-matching)			
	**Total**	**Urban**	**Children**	**Elderly**	**Total**	**Urban**	**Children**	**Elderly**
	(1)	(2)	(3)	(4)	(5)	(6)	(7)	(8)
Treatment ×Post	-0.1145***	-0.1422***	-0.0801	-0.3105**	-0.0928***	-0.0991***	-0.0639	-0.2876***
	(0.0302)	(0.0325)	(0.1411)	(0.1326)	(0.0121)	(0.0122)	(0.0625)	(0.0575)
Treatment					-0.0816***	-0.0819***	-0.0202	-0.0372
					(0.0205)	(0.0213)	(0.0576)	(0.0488)
Post	0.0471**	0.0100	0.0229	-0.2031	-0.0339***	-0.0582***	-0.0201	-0.0783*
	(0.0226)	(0.0261)	(0.0901)	(0.1598)	(0.0093)	(0.0074)	(0.0364)	(0.0465)
Cluster in group	No	No	No	No	Yes	Yes	Yes	Yes
Controls	Yes	Yes	Yes	Yes	Yes	Yes	Yes	Yes
Observation	410,258	304,000	6,408	5,978	222,554	167,066	3,532	3,394
R^2	0.0033	0.0030	0.0048	0.0305	0.0111	0.0092	0.0067	0.0204

Notes. The standard errors are clustered at the household-group level. Significance is at *** p<0.01, ** p<0.05, * p<0.1.

Supplementary Table 13. Estimations examining the effect of EDR assignment selection (comparison of results before and after the DTW-based matching method).

	EDR assignment selection (not DTW-matching)				EDR assignment selection (DTW-matching)			
	**Main effect**	**Main effect**	**Spillover effect**	**Spillover effect**	**Main effect**	**Main effect**	**Spillover effect**	**Spillover effect**
	(1)	(2)	(3)	(4)			(5)	
Treatment ×Post	-0.0153***	-0.0162***	-0.0018	-0.0034	-0.0153***	-0.0155***	-0.0018	-0.0024
	(0.0043)	(0.0043)	(0.0044)	(0.0044)	(0.0022)	(0.0023)	(0.0016)	(0.0016)
Treatment					-0.0315***	-0.0315***	-0.0224***	-0.0226***
					(0.0041)	(0.0039)	(0.0018)	(0.0017)
Post	-0.0352	0.0077	-0.0352***	0.0526**	-0.0352***	-0.0355***	-0.0352***	-0.0372***
	(0.0031)	(0.0180)	(0.0031)	(0.0249)	(0.0042)	(0.0090)	(0.0042)	(0.0093)
Cluster in group	No	No	No	No	No	Yes	No	Yes
Controls	No	Yes	No	Yes	Yes	Yes	Yes	Yes
Observation	410,258	410,258	378,114	378,114	410,258	410,258	378,114	378,114
F	215.74	84.55	134.6	59.09	70.81	61.98	116.5	79.73
R^2	0.0021	0.0028	0.0014	0.0021	0.0008	0.0085	0.0004	0.0082

Notes. This table shows the regression results before DTW matching (columns (1)-(4)) and after DTW matching (columns (5)-(8)), where columns (1), (2), (5), and (6) are the main effect of the EDR assignment selection and columns (3), (4), (7), and (8) are the spillover effect of the EDR. Standard errors in parentheses. Significance is at *** p<0.01, ** p<0.05, * p<0.1.

Supplementary Table 14. Estimations examining the effects of the EDR assignment selection among heterogeneous groups (comparison of results before and after the DTW-based matching method).

	EDR assignment selection (not DTW-matching)			EDR assignment selection (DTW-matching)		
Indicator —> EDR	**Urban**	**Children**	**Elderly**	**Urban**	**Children**	**Elderly**
	**(1)**	**(2)**	**(3)**	**(4)**	**(5)**	**(6)**
Treatment ×Post ×Indicator	-0.0290***	-0.0055	-0.0546**	-0.0284***	-0.0041	-0.0559**
	(0.0084)	(0.1173)	(0.0527)	(0.0036)	(0.0733)	(0.0264)
Treatment ×Post	0.0053	-0.0129	-0.0271	0.0063**	-0.0144	-0.0289*
	(0.0066)	(0.1127)	(0.0391)	(0.0027)	(0.0710)	(0.0165)
Indicator ×Post	0.0432***	0.1078	-0.0278	-0.0010	0.1053	-0.0327
	(0.0072)	(0.0903)	(0.0414)	(0.0094)	(0.0866)	(0.0391)
Indicator × Treatment				0.0113**	-0.0211	-0.0034
				(0.0044)	(0.0561)	(0.0224)
Treatment				-0.0401***	0.0016	-0.0165
				(0.0048)	(0.0527)	(0.0166)
Post	-0.0415**	-0.2818*	-0.1519	-0.0437***	-0.1436*	-0.0302
	(0.0191)	(0.1580)	(0.1128)	(0.0122)	(0.0838)	(0.0344)
Indicator				-0.1209***	0.0486	0.1540***
				(0.0245)	(0.0756)	(0.0440)
Cluster in group	No	No	No	Yes	Yes	Yes
Controls	Yes	Yes	Yes	Yes	Yes	Yes
Observation	410,258	7,466	10,322	410,258	7,466	10,322
F	70.98	2.290	9.409	51.94	2.692	11.24
R^2	0.0029	0.0076	0.0161	0.0094	0.0077	0.0147

Notes. This table shows the regression results before DTW matching (columns (1)-(3)) and after DTW matching (columns (4)-(6)). Standard errors in parentheses. Significance is at *** p<0.01, ** p<0.05, * p<0.1.

Supplementary Table 15. Estimations examining the effects of the EDR rebate coverage on electricity use (comparison of results before and after the DTW-based matching method).

	EDR rebate coverage (not DTW-matching)				EDR rebate coverage (DTW-matching)			
	**Total**	**Urban**	**Children**	**Elderly**	**Total**	**Urban**	**Children**	**Elderly**
	(1)	(2)	(3)	(4)	(5)	(6)	(7)	(8)
Treatment ×Post ×Indicator		-0.0672***	-0.0196	-0.1702**		-0.0699***	-0.0223	-0.1677**
		(0.0200)	(0.1933)	(0.0795)		(0.0184)	(0.1854)	(0.0762)
Treatment ×Post	-0.0940***	-0.0371**	-0.0462	-0.1067*	-0.0928***	-0.0303*	-0.0438	-0.1136**
	(0.0090)	(0.0180)	(0.1847)	(0.0648)	(0.0121)	(0.0180)	(0.1759)	(0.0546)
Indicator ×Post		0.0436***	0.1118	-0.0262		0.0039	0.1073	-0.0333
		(0.0080)	(0.0909)	(0.0421)		(0.0096)	(0.0869)	(0.0392)
Indicator ×Treatment						0.0365	-0.1564	-0.0243
						(0.0223)	(0.1705)	(0.0720)
Treatment					-0.0816***	-0.1113***	0.1311	-0.0139
					(0.0205)	(0.0303)	(0.1568)	(0.0589)
Post	0.0289	-0.0338	-0.3611*	-0.1919	-0.0339***	-0.0472***	-0.1488*	-0.0316
	(0.0254)	(0.0274)	(0.1843)	(0.1499)	(0.0093)	(0.0127)	(0.0852)	(0.0348)
Indicator						-0.1311***	0.0427	0.1534***
						(0.0248)	(0.0775)	(0.0445)
Cluster in group	No	No	No	No	Yes	Yes	Yes	Yes
Controls	Yes	Yes	Yes	Yes	Yes	Yes	Yes	Yes
Observation	222,554	222,554	4,124	5,814	222,554	222,554	4,124	5,814
F	68.54	56.92	1.613	13.24	63.12	48.68	1.855	11.87
R^2	0.0043	0.0045	0.0110	0.0343	0.0111	0.0121	0.0072	0.0209

Notes. This table shows the regression results before DTW matching (columns (1)-(4)) and after DTW matching (columns (5)-(8)). Standard errors in parentheses. Significance is at *** p<0.01, ** p<0.05, * p<0.1.

Supplementary Note 8. Sustainability of the Incentive-Based EDR Effect

To estimate the sustainability of the incentive-based EDR effect, we repeated our interventions over 6 treatment days in the summer (Supplementary Note 2.1; Supplementary Table 1). Recall that we determined the treatment days by day-ahead weather forecasts, and therefore, they were not necessarily consecutive. After excluding the trial with a small sample size (N = 805) for the first time, based on data on the electricity use of households that received invitation text messages consecutively in the last five EDR trials (The dates were the 26th of July 2019, 1st, 2nd, 8th, and 19th of August 2019), combined with the hourly meteorological data during the same period, the individual fixed effects model was expressed as follows:

$$Elec\_conservation_{it} = \delta_0 + \delta_1 Treat\_cnt_{it} + \gamma X_{it} + \alpha_i + \lambda_t + \varepsilon_{it} \quad \text{(Equation (S10))}$$

$$Elec\_conservation_{it} = \sum_{t \in T} (\beta_t EDR_{it} + \varphi_t Spillover_{it}) + \alpha_i + \lambda_t + \varepsilon_{it} \quad \text{(Equation (S11))}$$

where $Elec\_conservation_{it}$ is the electricity conservation in the declared on-peak times. $Treat\_cnt_{it}$ refers to the cumulative number of times participating (when estimating the main effects) or the number of invitations to participate (when estimating the spillover effects) in the EDR of household i in phase *t*. β_t and φ_t are the main effect of the EDR and the spillover effect in phase *t*, respectively. The term α_i is individual fixed effects, and it captures the time-invariant characteristics of household *i*. The term λ_t is time fixed effects, and it captures the time-variant characteristics of phase *t*. X_{it} represents the remaining control variables, including climate-related variables, namely, temperature, wind direction, wind speed, wind level, relative humidity, atmospheric pressure, vapor pressure, relative humidity and visibility. Our objective was to test how β_t and φ_t changed over the repeated interventions.

Supplementary Note 9. Robustness test

Note 9.1 parallel trend test

We used the electricity use data for the same declared on-peak times for six continuous days (8 pm-9.30 pm from August 15, 2019, to August 20, 2019) to generate interaction terms with the time dummy variables and treatment group dummy variables. The interaction terms were used as explanatory variables for the regression, and the coefficients reflect the difference between the treatment and control groups. The results showed that before the EDR, the interaction terms were not significant, which indicates that there was no significant difference between the treatment and the control group before the trial; that is, the parallel trend hypothesis was satisfied. Meanwhile, the coefficient of the interaction term on the treatment day (August 19, 2019) was significant and then again became nonsignificant on the next day (August 20, 2019). These findings indicate that the EDR had a significant effect only on the treatment day and that there were no systematic differences between the treatment and control groups before the trial.

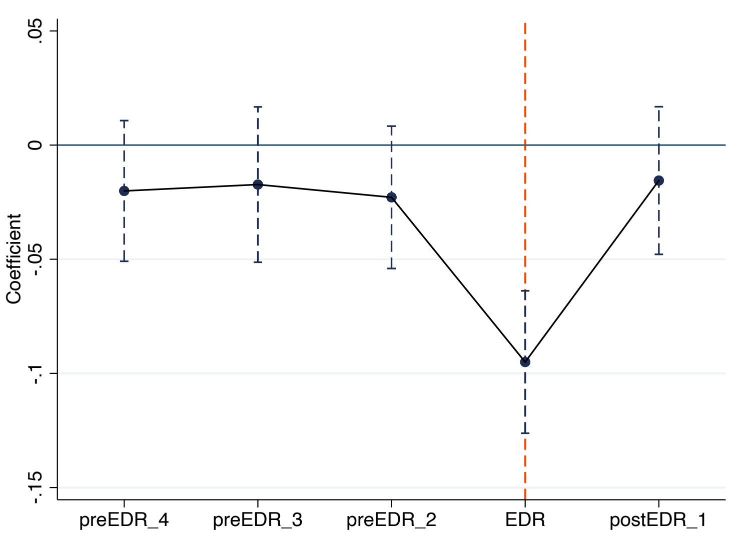

Supplementary Fig. 15 Parallel trend test. We used 4 days before and 1 day after the EDR for parallel trend testing. The results showed that the EDR was significant only on the treatment day, and the coefficients before and after the EDR were not significant. The day before EDR, named preEDR_1, was set as the baseline. The centers of the error bars are the values of the coefficients, which represent point estimates from the regressions and indicate the effect of EDR rebate coverage. The vertical lines represent the 95% confidence intervals of the study result, with each end of the line representing the boundaries of the confidence interval.

Note 9.2 Heckman two-step method test

Households participating in EDR may have self-selection bias. We adopted the Heckman two-step method as a robustness test. In the first stage, the selection equation is constructed, and some factors causing participants' potential motivation are selected as the exclusive constraint variables. In the second stage, the inverse Mills ratio is added for regression. The model is as follows:

$$pro\{exp_i = 1\} = \alpha_0 + \sum_{p\in P} \alpha_p z_i + X_{it} + \mu_i \qquad \text{(Equation (12))}$$

where exp_i refers to the possibility of participating in EDR for household i. z_i represents the exclusive constraint variable, i.e., frequency of activity enthusiasm (Act_enth), X_{it} represent the remaining control variables, including house area (*Area*), number of family members (*Person_cnt*), whether occupation is in the energy and environmental protection industries (*Ene_industry*), income (*Income*), electric vehicles (*Electric_car*), region (*Region*), community type (*D_leval*), number of air conditioners (*Aircon_cnt*), number of major appliances (*Lapp_cnt*), and average monthly electricity use (M_ave).

$$Elec_{it} = \beta_0 + \beta_1(Treatment_i * Post_t) + \gamma IMR_i + X_{it} + \upsilon_{it} \quad \text{(Equation (S13))}$$

where $Elec_{it}$ refers to the electricity use of household i in hour t. $Treatment_i$ is a dichotomous variable set to 1 if the household participated in the EDR trial and 0 if the household was in the control group. $Post_t$ is a dichotomous variable set to 1 if the date was the treatment day and 0 for the benchmark day. X_{it} represents the remaining control variables, including the air quality and climate-related variables, namely, wind direction, wind speed, wind level, relative humidity, vapor pressure, and relative humidity. The IMR obtained from the previous model is considered here, which means that the effect of exclusion restrictions can be ignored. The term υ_{it} is the error term.

We present the OLS, Heckman maximum likelihood, Heckman two-step and two-step split model results in Columns (1)-(5) of Supplementary Table 16. We found that Heckman's coefficient is significant (*coef. = -0.1239, p = 0.027*). The coefficients of other similar methods are also significant (*OLS coefficient. = -0.0844, p = 0.060; HeckMLE coef. = -0.1627, p = 0.033; Heck2SLS coef. = -0.1626, p = 0.033*). These results are consistent with the conclusion of the article.

Supplementary Table 16. Heckman two-step method test result.

	OLS<br>(1)	HeckMLE<br>(2)	Heck2SLS<br>(3)	First<br>(4)	Second<br>(5)
**main**					
Treatment	-0.0844*	-0.1627**	-0.1626**		-0.1239**
	(0.0449)	(0.0762)	(0.0762)		(0.0559)
wind_direction	-0.0002	0.0000	0.0000		-0.0002
	(0.0002)	(0.0004)	(0.0004)		(0.0002)
wind_speed	0.0235	0.0245	0.0244		0.0263
	(0.0227)	(0.0476)	(0.0476)		(0.0228)
relative_humidity	0.004	0.0099	0.0099		0.0046
	(0.0066)	(0.0137)	(0.0137)		(0.0066)
vapor_pressure	-0.0059	-0.0369	-0.0368		-0.0089
	(0.0203)	(0.0448)	(0.0448)		(0.0204)
m_ave	0.0030***	0.0027***	0.0027***		0.0030***
	(0.0001)	(0.0002)	(0.0002)		(0.0001)
city	0.0532	-0.0033	-0.0034		0.0585*
	(0.0346)	(0.0740)	(0.0740)		(0.0349)
d_level	0.0021	0.016	0.016		0.0022
	(0.0119)	(0.0234)	(0.0234)		(0.0119)
Act_enth				1.5462***	
				(0.0485)	
Area				0.0158	
				(0.0168)	
Person_cnt				0.0030	
				(0.0069)	
Ene_industry				0.3974***	
				(0.1020)	
Income				-0.016	
				(0.0137)	
Electric_car				-0.1663**	
				(0.0795)	
Region				0.0277	
				(0.0280)	
D_leval				0.0636**	
				(0.0249)	

Aircon_cnt	0.0856*
	(0.0519)
Lapp_cnt	0.0452
	(0.0444)

Continue. Supplementary Table 16. Heckman two-step method test result

	OLS (1)	HeckMLE (2)	Heck2SLS (3)	First (4)	Second (5)
M_ave				-0.0014***	
				(0.0002)	
IMR					0.0319
					(0.0270)
_cons	0.2662	1.0658**	1.0670**	-1.9409***	0.2977
	(0.1830)	(0.4631)	(0.4633)	(0.1833)	(0.1849)
att					
Act_enth		1.5548***	1.5543***		
		(0.0488)	(0.0489)		
Area		0.0184	0.0187		
		(0.0169)	(0.0169)		
Person_cnt		0.0016	0.0013		
		(0.0070)	(0.0069)		
Ene_industry		0.4129***	0.4164***		
		(0.1031)	(0.1028)		
Income		-0.0137	-0.0138		
		(0.0138)	(0.0138)		
Electric_car		-0.1597**	-0.1575**		
		(0.0799)	(0.0798)		
Region		-0.0253	-0.0261		
		(0.0323)	(0.0323)		
D_leval		0.0378	0.0378		
		(0.0258)	(0.0258)		
Aircon_cnt		0.0845	0.0838		
		(0.0519)	(0.0520)		
Lapp_cnt		0.0547	0.0523		
		(0.0447)	(0.0445)		
M_ave		-0.0014***	-0.0014***		
		(0.0002)	(0.0002)		
View_freq		1.5548***	1.5543***		
		(0.0488)	(0.0489)		
city		-0.1629***	-0.1632***		
		(0.0357)	(0.0357)		
d_level		0.026	0.0264		
		(0.0202)	(0.0202)		
_cons		-1.4177***	-1.4066***		
		(0.2182)	(0.2174)		
/					
athrho		-0.0367			
		(0.0661)			
lnsigma		0.0037			
		(0.0198)			
mills			-0.0388		
			(0.0687)		
N	5528	5528	5528	5528	5528
adj. R^2	0.14				0.14
pseudo R^2				0.211	

Notes. The standard errors are clustered at the household level. Significance is at *** p<0.01, ** p<0.05, * p<0.1.

Note 9.3 Placebo test

Supplementary Table 17. Estimations examining the effect of EDR assignment selection (intent-to-treat, placebo test).

	EDR assignment selection		Heterogeneous effect		
	Main effect	Spillover effect	Urban	Children	Elderly
	(1)	(2)	(3)	(4)	(5)
Treatment ×Post ×Indicator			0.0199	-0.0241	0.0124
			(0.0203)	(0.0441)	(0.0213)
Treatment ×Post	0.0200	0.0094	0.0075	0.0157	-0.0021
	(0.0228)	(0.0110)	(0.0108)	(0.0434)	(0.0161)
Indicator ×Post			-0.1572	0.0297	0.0903**
			(0.1316)	(0.0711)	(0.0406)
Indicator × Treatment			-0.0075	-0.0438	-0.0495**
			(0.0053)	(0.0404)	(0.0195)
Treatment	-0.0343*	-0.0272	-0.0295	0.0406	0.0126
	(0.0205)	(0.0210)	(0.0223)	(0.0404)	(0.0122)
Post	-0.1518	-0.1565	-0.0754	-0.2803***	-0.2774***
	(0.1491)	(0.1518)	(0.0623)	(0.0804)	(0.0925)
Indicator			-0.0630	0.1282*	0.0894**
			(0.0481)	(0.0698)	(0.0396)
Cluster in group	Yes	Yes	Yes	Yes	Yes
Controls	Yes	Yes	Yes	Yes	Yes
Observation	297,230	273,211	297,230	5,869	8,224
F	46.75	45.56	34.37	4.30	5.40
R^2	0.0001	0.0000	0.0001	0.0203	0.0251

Notes. This table reports the estimated coefficients and cluster-robust standard errors (in parentheses) in Equations (1) and (2) (see "Methods"). The dependent variable in all columns is electricity usage during counterfactual on-peak times. Columns (1) and (3-5) estimate the effect of assignment selection comparing the average outcome for households selected in the random assignment (EDR group and no-reply group, which we called assignment winners) to the average outcome for control households (no-notification group those not selected by the assignment) among heterogeneous groups. Column (2) compares the difference between the no-reply group and the no-notification group. The standard errors are clustered at the household-group level. Significance is at *** p<0.01, ** p<0.05, * p<0.1.

Supplementary Table 18. Estimations examining the effects of EDR rebate coverage (IV two-stage approach and DTW matching method, placebo test)

	EDR rebate coverage (IV two-stage)				EDR rebate coverage (DTW-matching)			
	Total	Urban	Children	Elderly	Total	Urban	Children	Elderly
	(1)	(2)	(3)	(4)	(5)	(6)	(7)	(8)
Treatment ×Post ×Indicator		0.1486*	-0.0734	0.0221		0.1307	-0.0660	0.0125
		(0.0897)	(0.1550)	(0.0740)		(0.1117)	(0.1571)	(0.0213)
Treatment ×Post	0.2600	-0.0446**	0.0420	-0.0197	0.0658	-0.0359*	0.0343	-0.0022
	(0.7443)	(0.0199)	(0.1472)	(0.0580)	(0.0809)	(0.0200)	(0.1525)	(0.0160)
Indicator ×Post		-0.2230*	0.0463	0.1017***		-0.1389	0.0275	0.0901**
		(0.1268)	(0.0515)	(0.0286)		(0.1177)	(0.0710)	(0.0407)
Indicator ×Treatment						0.0561	-0.1417	-0.0494**
						(0.0349)	(0.1341)	(0.0195)
Treatment					-0.0582***	-0.1052**	0.1432	0.0127
					(0.0195)	(0.0409)	(0.1297)	(0.0121)
Post	-0.0843	0.0622**	-0.1974***	-0.1511***	-0.1568	-0.0920	-0.3012***	-0.2706***
	(0.0597)	(0.0313)	(0.0649)	(0.0448)	(0.1459)	(0.0708)	(0.0974)	(0.0714)
Indicator						-0.0811**	0.1183*	0.0894**
						(0.0350)	(0.0704)	(0.0396)
Cluster in group	No	No	No	No	Yes	Yes	Yes	Yes
Controls	Yes	Yes	Yes	Yes	Yes	Yes	Yes	Yes
Observation	297,230	297,230	5,869	8,224	164,119	164,119	3,244	8,224
R^2	0.0000	0.0001	0.0074	0.0120	0.0001	0.0001	0.0190	0.0251

Notes. This table reports the estimated coefficients and cluster-robust standard errors (in parentheses) in Equations (1) and (2) (see "Methods"). The dependent variable in all columns is electricity usage during counterfactual on-peak times. Columns (1-4) use a standard instrumental-variable approach with assignment selection as an instrument to estimate EDR rebate coverage among heterogeneous groups. Column (5-8) compares the difference between the EDR group and the no-notification group by using matching methods. The standard errors are clustered at the household-group level. Significance is at *** p<0.01, ** p<0.05, * p<0.1.

Supplementary Note 10. Conversion standard for power and electricity use of household appliances

To avoid excessive infringement on the privacy of households, we installed smart sockets in only 15 households participating in the EDR trial with the consent of the households to collect use data of household appliances (such as refrigerators, air conditioners, washing machines, televisions, and water heaters) in minutes. Combined with data of household appliances sold on e-commerce platforms, we estimated the power of household appliances and used the case study method to analyze what electricity saving behaviors households may take in response to the EDR. The sample includes the use data of 58 household appliances in 15 households from July 2019 to September 2019 and the power of some models of household appliances.

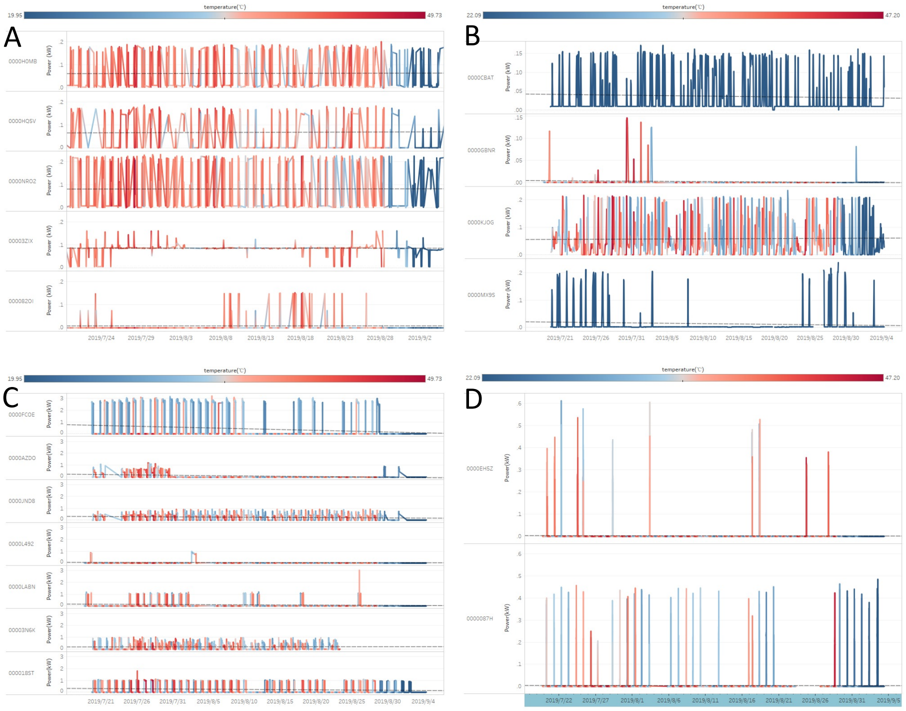

Supplementary Fig. 16 Working power of common household appliances. A-D are working power data for refrigerators, air conditioners, washing machines, and televisions from July 2019 to September 2019, and the collection frequency is minute level. The ordinate is the working power of each DK (model code of the appliances), the color represents the hourly temperature of the day, and the color from blue to red indicates that the temperature is from low to high.

Supplementary Table 19. Measured power of various types of household appliances

**Household appliances**	**DK**	**Power(kW)**	**Household appliances**	**DK**	**Power(kW)**
**Air conditioner (bedroom)**	000018ST	0.9646	Refrigerator	000003ZIX	0.1146
	00003N6K	0.9086		000082OI	0.1186
	0000AZDO	0.9695		0000NRO2	0.1004
	0000JND8	0.8570		0000HOMB	0.1806
	0000L492	0.8653		0000HQ5V	0.1639
	0000LABN	0.9951	Television	0000CBAT	0.1491
**Water heater**	00006XGB	0.1979		0000GBNR	0.1394
	0000HKMI	1.8009		0000KJOG	0.2101
	0000HK67	1.3021		0000MX9S	0.1893
**Washing machine**	0000087H	0.2303	Air conditioner (living room)	0000FCOE	2.7183
	0000EH5Z	0.2679			

Notes. DK represents the model code of the household appliances.

Supplementary Table 20. Conversion standard for power and electricity of household appliances

	AC (bedroom) 1102.5W	AC (living room) 2205W	refri. 150W	WM 300W	WH 2000W	TV 180W	MO 1200W	VC 800W
Total households (0.1145 kWh)	6.2	3.1	45.8	22.9	3.4	38.2	5.7	8.6
Urban households (0.9910 kWh)	5.4	2.7	39.6	19.8	3.0	33.0	5.0	7.4
Rural households (0.5630 kWh)	3.1	1.5	22.5	11.3	1.7	18.8	2.8	4.2
Households with elderly (0.1638 kWh)	8.9	4.5	65.5	32.8	4.9	54.6	8.2	12.3

Notes. The meanings of the abbreviations in the table are as follows: AC, air conditioner; refri., refrigerator; WM, washing machine; WH, water heater; TV, television; MO, microwave oven; VC, vacuum cleaner. The air conditioner (bedroom) is generally 1.5 horsepower, and one horsepower is approximately 735 W; when the air conditioner is in cooling mode, if the set temperature is lowered by one degree, the 1.5 horsepower air conditioner will use more than 0.3 kWh for five hours continuously. The unit of the numbers in the table is minutes.

We selected two households (M, N) and analyzed the specific electricity-saving behaviors they took in response to the EDR via the case study method.

For example, Supplementary Fig. 17 shows the working power of household M's main household appliances (air conditioner, washing machine, television) during the EDR period. We found that the household turned on the air conditioner until the EDR ended, and there was no significant change in washing machine and television usage.

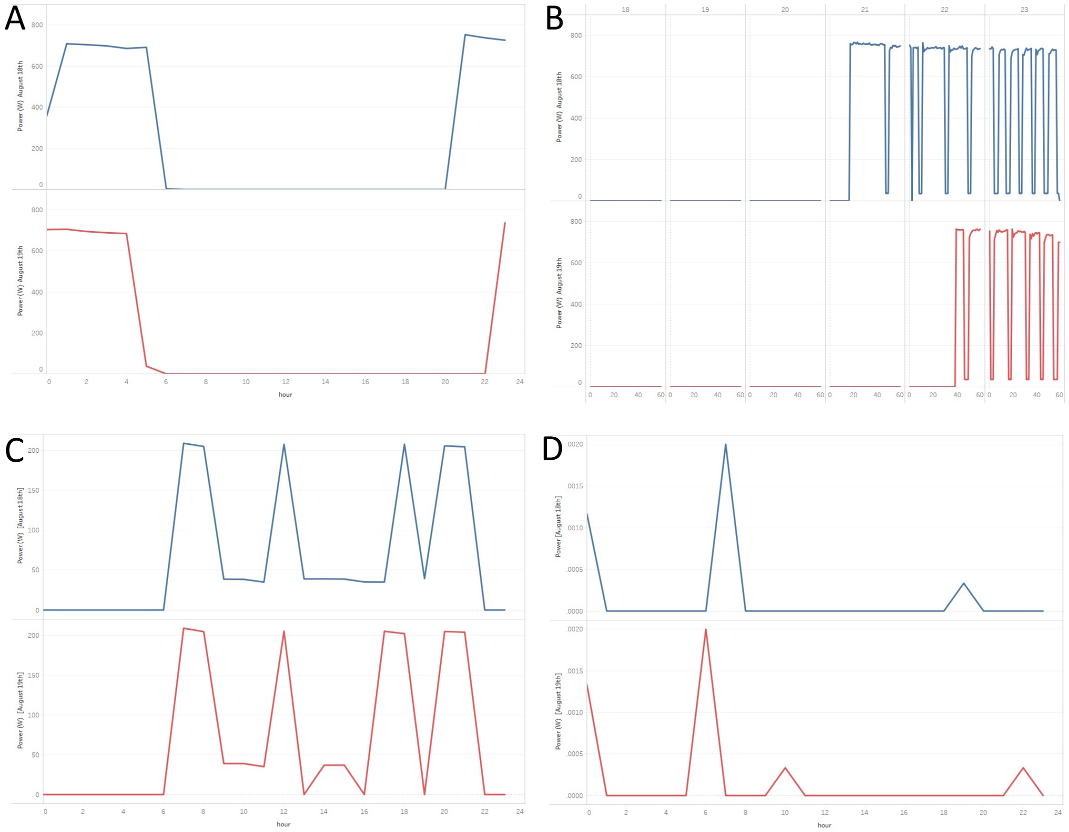

Supplementary Fig. 17 Working power of household appliances for household M. A, C, D are working power data for the air conditioner, television, and washing machine on the benchmark day (blue lines) and treatment day (red lines). The abscissa is from 0:00 to 24:00, and the ordinate is power (W). **B** zooms in on **A** from 18:00 to 23:00.

Supplementary Fig. 18 shows the working power of household N’s main household appliances (air conditioner, washing machine, television) during the EDR period. We found that the household turned off the washing machine during the EDR period, and there was no significant change in refrigerator or television usage.

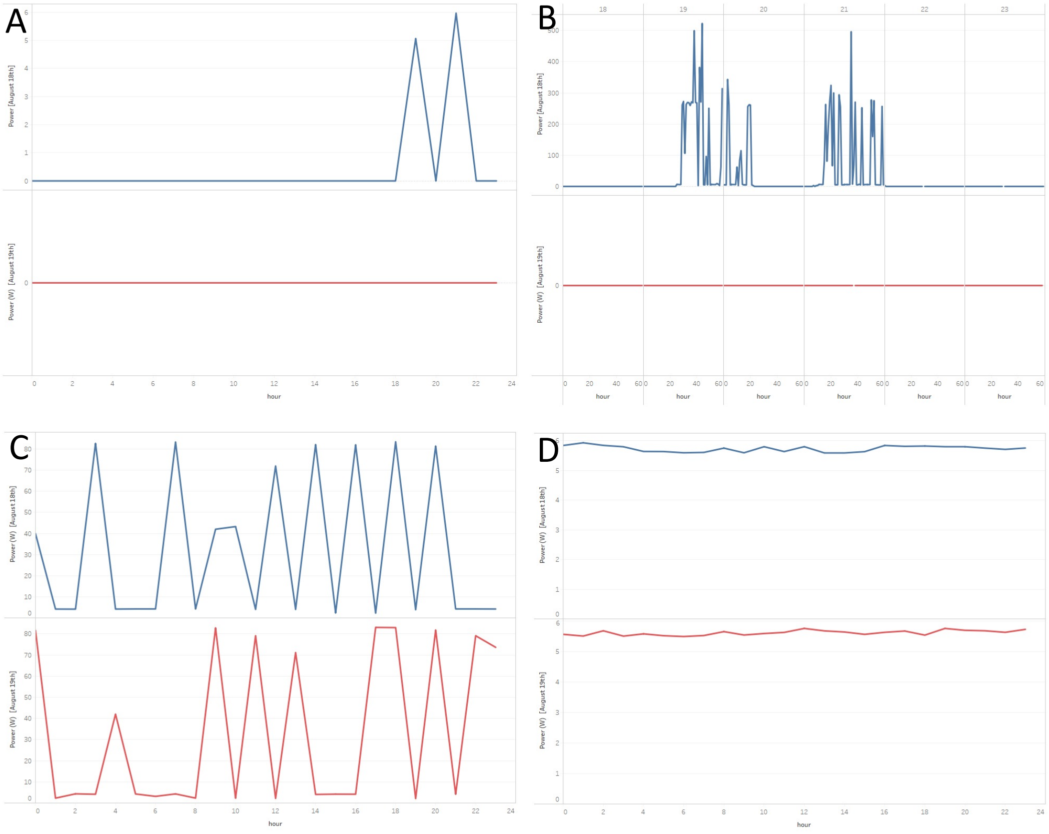

Supplementary Fig. 18 Working power of household appliances for household N. A, C, D are working power data for the washing machine, refrigerator, and television on benchmark days (blue lines) and treatment days (red lines). The abscissa is from 0:00 to 24:00, and the ordinate is power (W). **B** zooms in **A** from 18:00 to 23:00.

Supplementary Note 11. Heterogeneous treatment effect analysis across different electricity price

The pilot provinces in Southwest China adopt the increasing block price for residents (Supplementary Fig. 19). The incentive policy of ¥1/kWh is equivalent to reducing the current electricity price by 11.1%-66.7%. In the increasing block rate, there are three tiers of marginal price (MP). The marginal price is ¥0.6/kWh when the cumulative annual electricity consumption **in the current year** y ≤ 2160 kWh, ¥0.65/kWh when 2160 kWh < y ≤ 4200 kWh, ¥0.9/kWh when y > 4200 kWh. Consumers may make the optimal decision to maximize their net benefits. As demonstrated in Figure 1, from the first block to the last, marginal prices experience greater price change than average prices (AP).

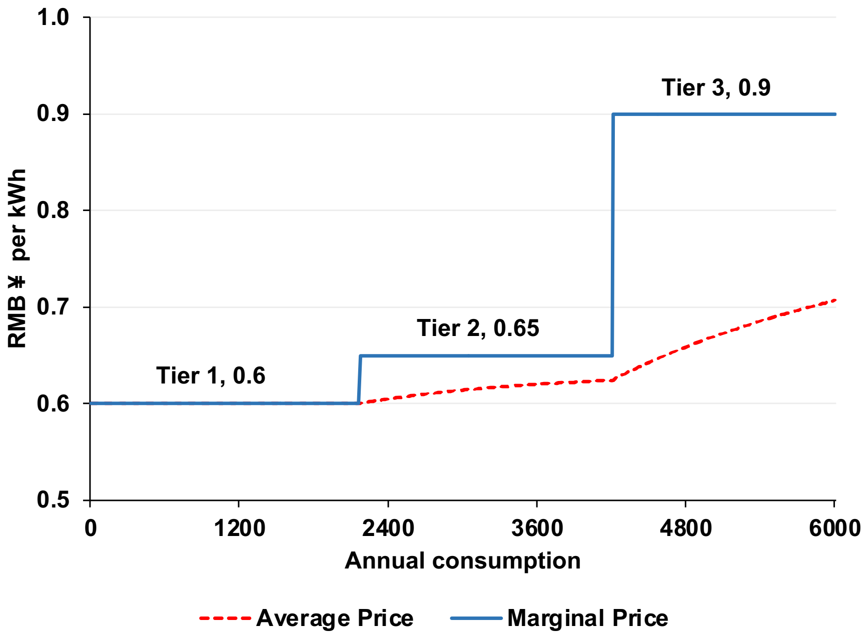

Supplementary Fig. 19 An example of cross-sectional price variation in nonlinear electricity pricing

Regression model with MP and AP

We use the DID fixed effect regression and an instrumental variable (IV) approach to include the effects of electricity prices. The identification assumption is that confounding factors such as underlying distributional changes in consumption are not systematically different across the random assignment of the treatment, which we use as the IV.

$$Elec_{it} = \beta_0 + \beta_1(Treatment_i \times Post_t) + \beta_2 MP_{i,m} + \beta_3 AP_{i,m} + \theta_i + \psi_t + X_{it} + \varepsilon_{it}$$

(Equation (S14))

with the instrument variable $IV\_assignment_{it}$, by fitting two-stage least squares regressions (with assignment selection as an instrument for EDR rebate coverage). We add the average price ($AP_{i,m}$) and marginal price ($MP_{i,m}$) to the model as the control

variables. Since the time granularity in the model is at the hour level and the pilot provinces implement an increasing block price based on the annual cumulative electricity consumption, the price variables are thus absorbed by the fixed effects. Table S1 shows that the results of the two-way fixed effect DID model and the IV two-stage model are consistent and have a significant electricity-saving effect. In order to intuitively estimate the impact of electricity prices, we have added additional analyses.

Supplementary Table 21. Regression model with MP and AP

	(1) EDR rebate coverage (DID)	(2) EDR rebate coverage (IV two-stage)
Treatment ×Post	-0.1039***	-0.1280***
	(0.0093)	(0.0323)
Household Fixed Effect	YES	YES
Time Fixed Effect	YES	YES
Controls	YES	YES
Observation	375,850	375,850
R-squared	0.0016	0.0037

Notes: This table reports the estimated coefficients and cluster-robust standard errors (in parentheses). The dependent variable in all columns is electricity usage during on-peak times. The standard errors are clustered at the household level. Significance is at *** p<0.01, ** p<0.05, * p<0.1.

Heterogeneous treatment effect analysis

In order to focus on examining varying treatment effects across different electricity prices, we build a difference-in-difference-in-difference (DDD)-like model, and we are interested in the coefficient of the interaction term multiplied by the electricity price variable ($Treatment_i \times Post_t \times \boldsymbol{Price_{i,m}}$). Different from the standard DDD, the electricity price here is not a dummy variable, but an increasing block price (0.6, 0.65, 0.9) and an average price (this is a continuous variable). We consider three forms of marginal electricity price and average electricity price: one-month lag ($\boldsymbol{Lag1.}MP_{i,m}$, $\boldsymbol{Lag1.AP_{i,m}}$), six-month average (accumulated from six months before the pilot, $Accum\boldsymbol{Mon.MP_{i,m}}$, $Accum\boldsymbol{Mon.AP_{i,m}}$), four-year average (accumulated from January of 2016 to the month of the pilot, $Accum\boldsymbol{Year.MP_{i,m}}$, $Accum\boldsymbol{Year.AP_{i,m}}$).

$$Elec_{it} = \beta_0 + \beta_1(Treatment_i \times Post_t \times \boldsymbol{Price_{i,m}}) + \beta_2(Treatment_i \times \boldsymbol{Price_{i,m}}) + \beta_3(Post_t \times \boldsymbol{Price_{i,m}}) + \beta_4(Treatment_i \times Post_t) + \beta_5 Treatment_i + \beta_6 Post_t + \beta_7 \boldsymbol{Price_{i,m}} + \theta_i + \psi_t + X_{it} + \varepsilon_{it} \qquad \text{(Equation (S15))}$$

where $\boldsymbol{Price_{i,m}}$ is replaced by $\boldsymbol{MP_{i,m}}$ and $\boldsymbol{AP_{i,m}}$ respectively in subsequent analysis. Note that this is a full-form model, and some variables will be absorbed by fixed effects.

Supplementary Table 22. Heterogeneous treatment effect analysis across different electricity price

	Lag1		*AccumMon*		*AccumYear*	
	(1)	(2)	(3)	(4)	(5)	(6)
Treatment ×Post ×$\boldsymbol{MP_{i,m}}$	-0.5413**		-0.8214***		-0.3161**	
	(0.2535)		(0.3111)		(0.1543)	
Treatment ×Post ×$\boldsymbol{AP_{i,m}}$		-0.6489**		-2.0680**		-1.2648*
		(0.2892)		(0.8807)		(0.7388)
Household Fixed Effect	YES	YES	YES	YES	YES	YES
Time Fixed Effect	YES	YES	YES	YES	YES	YES

Controls	YES	YES	YES	YES	YES	YES
Observation	375,850	375,850	375,850	375,850	209,706	209,706
R-squared	0.0017	0.0017	0.0017	0.0017	0.0015	0.0016

Notes: This table reports the estimated coefficients for the key interaction term only and cluster-robust standard errors (in parentheses). The dependent variable in all columns is electricity usage during on-peak times. The standard errors are clustered at the household level. Significance is at *** p<0.01, ** p<0.05, * p<0.1.

The results in Supplementary Table 21 show that households with higher electricity prices tend to respond more actively to EDR, resulting in greater electricity savings during peak hours.

Sub-group regression analysis across different electricity marginal price

Furthermore, on the basis of Equation (S14), we examine the effect of emergency demand respond (EDR) for households across varying electricity marginal prices. Our results (Supplementary Table 23) indicate that EDR has more substantial effects on households with higher marginal electricity prices (P < 0.001), which is consistent with the conclusion in Heterogeneous treatment effect analysis.

Supplementary Table 23. Group regression analysis across different electricity marginal price

	EDR rebate coverage (FE)			EDR rebate coverage (IV two-stage)		
	(1) Tier 1 ≤ 2160 kWh	(2) Tier 2 >2160 kWh, ≤ 4200 kWh	(3) Tier 3 >4200 kWh	(4) Tier 1 ≤ 2160 kWh	(5) Tier 2 >2160 kWh, ≤ 4200 kWh	(6) Tier 3 >4200 kWh
Treatment× Post	-0.0803***	-0.1739***	-0.2007***	-0.1001***	-0.1971***	-0.3126*
	(0.0089)	(0.0259)	(0.0813)	(0.0333)	(0.0855)	(0.2600)
Household Fixed Effect	YES	YES	YES	YES	YES	YES
Time Fixed Effect	YES	YES	YES	YES	YES	YES
Controls	YES	YES	YES	YES	YES	YES
Observation	302,378	62,618	10,854	302,378	62,618	10,854
R-squared	0.0016	0.0025	0.0023	0.0042	0.0040	0.0026

Notes: This table reports the estimated coefficients and cluster-robust standard errors (in parentheses). The dependent variable in all columns is electricity usage during on-peak times. The standard errors are clustered at the household level. Significance is at *** p<0.01, ** p<0.05, * p<0.1.

Supplementary Note 12. Heterogeneous treatment effect analysis across different types of housing occupancy

In the pilot study, we obtained the renter/owner information from the survey. Among those who returned valid surveys, 6462 households are self-owned and 1312 are tenants. Whether people live in their own house may lead to differences when they are choosing to participate (or not) in the EDR pilot. We adopt an instrumental variable two-stage approach to estimate the effect of the treatment - not just assignment to treatment - that can account for noncompliance. To adjust for noncompliance, one can use the random assignment to treatment as an instrument for treatment receipt **since the initial assignment was random** (The identification strategy is referenced from *Science*). We estimate the effects of EDR rebate coverage by fitting two-stage least squares regressions (with assignment selection as an instrument for EDR rebate coverage). The coefficient of *Treatment×Post* identifies the causal effect of EDR rebate coverage among the subset of households who participated in the EDR upon

winning the assignment but who would not participate in EDR without winning the assignment (i.e., the compliers, find more details in Supplementary Note 6). We now also performed the above IV analysis on the subsample of survey respondents who reported self-owned and tenant information.

Supplementary Table 24 shows that in the subsample, the results of the two-way fixed effect DID model and the results of the IV two-stage model are consistent and have a significant electricity-saving effect.

Supplementary Table 24. EDR rebate coverage on the subsample of survey respondents who reported self-owned and tenant information

	(1) EDR rebate coverage (DID)	(2) EDR rebate coverage (IV two-stage)
Treatment ×Post	-0.1388***	-0.1719**
	(0.0300)	(0.0760)
Household Fixed Effect	YES	YES
Time Fixed Effect	YES	YES
Controls	YES	YES
Observation	15,548	15,548
R-squared	0.0035	0.0060

Notes: This table reports the estimated coefficients and cluster-robust standard errors (in parentheses). The dependent variable in all columns is electricity usage during on-peak times. The standard errors are clustered at the household level. Significance is at *** p<0.01, ** p<0.05, * p<0.1.

We do not have exact information to know whether the utility bill of each household is included in the rent. However, feedback from the leasing company and tenants suggests that almost all lease contracts require tenants to pay the utility bill in addition to rent. Therefore, we assume that the utility bill is not covered by the rent and build the following model based on this common situation:

$$Elec_{it} = \beta_0 + \beta_1(Treatment_i \times Post_t \times \boldsymbol{Htype_i}) + \beta_2(Treatment_i \times \boldsymbol{Htype_i}) + \beta_3(Post_t \times \boldsymbol{Htype_i}) + \beta_4(Treatment_i \times Post_t) + \beta_5 Treatment_i + \beta_6 Post_t + \beta_7 \boldsymbol{Htype_i} + \theta_i + \psi_t + X_{it} + \varepsilon_{it} \quad (Equation\ (S16))$$

Our findings indicate that EDR has a more significant effect on owner's electricity savings behavior during peak hours compared to tenants (coef. = -0.1472, p = 0.028, Supplementary Table 25).

Supplementary Table 25. Regression results by house type

**Regression estimates**	**Coef. ($\boldsymbol{T_i \times P_t}$)**	**SE**	***R-squared***	**Number of observations**
**Interactions in Regression**				
Treatment ×Post ×Htype	-0.1472**	0.0669	0.0040	15,548
**Group regression**				
Treatment ×Post in Owner group	-0.1592***	0.0335	0.0042	12,926
Treatment ×Post in tenants group	-0.0103	0.0581	0.0051	2,624
Household Fixed Effect	YES			
Time Fixed Effect	YES			
Controls	YES			

Note: Standard errors are clustered at the household level. Significance is at *** p<0.01, ** p<0.05, * p<0.1.

Then, we also conducted a sub-group regression analysis to estimate the differences between the two types of housing occupancy. Our results show that the EDR measure led to a significant load reduction in households living in their own houses (coef. = -0.1592, p < 0.001, Table 5). However, tenants did not experience a significant

load reduction despite also being responsible for paying utility bills. There are several possible reasons as follows: 1). Although we assume that the utility bills are not included in the rent according to the normal scenario, there may be exceptions where the tenant and the landlord have agreed that the rent includes the utility bill. 2). The insufficient sample size of tenants may have affected the estimation results. 3). Short-term tenants may not be interested in such policy information related to energy-saving programs. 4). There is a longstanding energy equipment negotiation problem between landlords and tenants. Landlords are reluctant to install or update energy-saving equipment due to economic cost, which makes it difficult for tenants to effectively participate in energy-saving programs.

Supplementary Note 13. Appendix

Questionnaire on residential energy consumption for the "Electricity Demand Response" project (Part of items)
User id (smart meter number):
Contact details:
Inspector:
Investigation date:

1. Living status ()
A. Own house B. Rent the house C. Other

2. The area of permanent residence is () square meters.
A. Less than 50 B. 50-70 C. 70-90 D. 90-110 E.110-130 F. 130-150 G. 150-200 H. 200-300 I. More than 300

3. Age of permanent residents (fill in the number of members in the corresponding age stage)
0-3 years old: ___ 4-18 years old: ___ 19-22 years old: ___ 23-30 years old: ___ 31-45 years old: ___
46-60 years old: ___ 61-70 years old: ___71-80 years old: ___ Over 80 years old: ___

4. The industry that family members are engaged in ()
A. Agriculture B. Manufacturing industry C. Real Estate/Construction D. Financial industry
E. Service Industry F. Education/Media/Entertainment G. Trade/Tourism H. Energy/Minerals/Environmental Protection
I. Transportation/Logistics J. Health/Sports K. IT/Communications/Internet L. Government/non-profit organization
M. Other ____

5. Does your family use electric water heater for showering?
A. Yes
B. No

6. Family annual comprehensive after-tax income ()
A. Below 50,000 RMB
B. 50,000-100,000 RMB or less
C. 10-18 million RMB
D. 18-36 million RMB
E. 360,000 RMB or more
F. Confidential

7. Does your family have an electric car?
A. Yes
B. No

8. The number of the following household appliances in your home:
air conditioners: ___ refrigerator: ___ washing machine: ___ water heater: ___

9. How do you follow or participate in community activities?
A. Once a month
B. Once every three months
C. Once every six months

10. If you find that your neighbor's electricity consumption this month is less than yours, will it affect your electricity consumption next month?
A. Affected by this, my electricity consumption may increase next month
B. Affected by this, my electricity consumption may decrease next month
C. I will not be affected in any way

11. If you are provided with a paid return, would you be willing to reduce electricity consumption during peak hours to alleviate the power gap?
A. Yes B. No

12. During peak hours of electricity consumption, are you willing to let us intelligently control household appliances to achieve better energy-saving effects ()
A. Yes B. No

References

1. Tol R. The economic effects of climate change. *J Econ Perspect.* 23(2), 29-51 (2009).
2. Rose S, et al. Understanding the social cost of carbon: A technical assessment. *EPRI Technical Update Report* (2014).
3. Segal, M., Shafir, H., Mandel, M., Alpert, P., & Balmor, Y. Climatic-related evaluations of the

summer peak-hours electric load in israel. *J Appl Meteorol*. 31(12), 1492-1498 (1992).
4. Thatcher, Marcus J. Modelling changes to electricity demand load duration curves as a consequence of predicted climate change for Australia. *Energy*. 32(9), 1647-1659 (2017).
5. Li Y , Pizer W A , Wu L. Climate change and residential electricity consumption in the Yangtze River Delta, China. *Proc Natl Acad Sci*. 116(2), 472-477 2019.
6. Hui Zhou, Wenjie Niu, Hong Ji. Analysis of residents' electricity consumption in summer under the influence of various factors. *North China Electric Power.* 21(3), 12-14 (2003)
7. Zhihua Wang, Xiaomei Yang, Yang Li. Research on the relationship between temperature and typical seasonal power load. *Electr Power Autom Eq*. 22(3), 16-18 (2002)
8. Deschenes O, Greenstone M. Climate change, mortality, and adaptation: Evidence from annual fluctuations in weather in the US. *Am Econ J Appl Econ*. 3(4), 152-185 (2011).
9. Auffhammer M, Aroonruengsawat A. Simulating the impacts of climate change, prices and population on California's residential electricity consumption. *Clim Change*. 109(S1), 191-210 (2011).
10. Deschenes O, Greenstone M. Climate change, mortality, and adaptation: Evidence from annual fluctuations in weather in the US. *Am Econ J Appl Econ*. 3(4), 152-185 (2011).
11. Auffhammer M, Aroonruengsawat A. Simulating the impacts of climate change, prices and population on California's residential electricity consumption. *Clim Change*. 109(S1), 191-210 (2011).
12. Wang Z , Hong T , Li H , Piette, M. A. Predicting city-scale daily electricity consumption using data-driven models. *Advances in Applied Energy.* 26(2), (2021).
13. Waite, M., Cohen, E., Torbey, H., Piccirilli, M., Tian, Y., & Modi, V. Global trends in urban electricity demands for cooling and heating. *Energy*. 127, 786-802 (2017).
