# Supplementary material for: Incentive based emergency demand response effectively reduces peak load during heatwave without harm to vulnerable groups
Source: Nat Commun. 2023 Oct 4;14:6202. doi: 10.1038/s41467-023-41970-8 (PMC10550920; doi:10.1038/s41467-023-41970-8)
Supplement: Supplementary file 2 — Reporting Summary [file 41467_2023_41970_MOESM2_ESM.pdf]

# nature portfolio

Corresponding author(s): Bo Wang, Yueming (Lucy) Qiu, Bin Zhang, Bin Lu

Last updated by author(s): Jun 1, 2023

# Reporting Summary

Nature Portfolio wishes to improve the reproducibility of the work that we publish. This form provides structure for consistency and transparency in reporting. For further information on Nature Portfolio policies, see our Editorial Policies and the Editorial Policy Checklist.

## Statistics

For all statistical analyses, confirm that the following items are present in the figure legend, table legend, main text, or Methods section.

| n/a | Confirmed | |
|---|---|---|
| ☐ | ☒ | The exact sample size (*n*) for each experimental group/condition, given as a discrete number and unit of measurement |
| ☐ | ☒ | A statement on whether measurements were taken from distinct samples or whether the same sample was measured repeatedly |
| ☐ | ☒ | The statistical test(s) used AND whether they are one- or two-sided<br>*Only common tests should be described solely by name; describe more complex techniques in the Methods section.* |
| ☐ | ☒ | A description of all covariates tested |
| ☐ | ☒ | A description of any assumptions or corrections, such as tests of normality and adjustment for multiple comparisons |
| ☐ | ☒ | A full description of the statistical parameters including central tendency (e.g. means) or other basic estimates (e.g. regression coefficient) AND variation (e.g. standard deviation) or associated estimates of uncertainty (e.g. confidence intervals) |
| ☐ | ☒ | For null hypothesis testing, the test statistic (e.g. *F*, *t*, *r*) with confidence intervals, effect sizes, degrees of freedom and *P* value noted<br>*Give P values as exact values whenever suitable.* |
| ☒ | ☐ | For Bayesian analysis, information on the choice of priors and Markov chain Monte Carlo settings |
| ☐ | ☒ | For hierarchical and complex designs, identification of the appropriate level for tests and full reporting of outcomes |
| ☐ | ☒ | Estimates of effect sizes (e.g. Cohen's *d*, Pearson's *r*), indicating how they were calculated |

*Our web collection on statistics for biologists contains articles on many of the points above.*

## Software and code

Policy information about availability of computer code

| | |
|---|---|
| Data collection | We conducted six emergency demand response pilots based on monetary rewards in southwestern China during heatwave. Before the pilots we installed high-speed power line communication (HPLC) smart meters in the relevant regions to collect high-frequency electricity consumption data.<br>At the same time, we randomly conducted a survey to obtain demographic information and ask whether they lived with children or elderly individuals. |
| Data analysis | The software we used as follows: Python3.6.5, STATA 15.0, Microsoft Excel 2019. |

For manuscripts utilizing custom algorithms or software that are central to the research but not yet described in published literature, software must be made available to editors and reviewers. We strongly encourage code deposition in a community repository (e.g. GitHub). See the Nature Portfolio guidelines for submitting code & software for further information.

## Data

Policy information about availability of data

All manuscripts must include a data availability statement. This statement should provide the following information, where applicable:

- Accession codes, unique identifiers, or web links for publicly available datasets
- A description of any restrictions on data availability
- For clinical datasets or third party data, please ensure that the statement adheres to our policy

Aggregating data (after removing personal identifiers) are provided on GitHub at https://github.com/BinLu-leo/Emergency-Demand-Response-effect.

## Research involving human participants, their data, or biological material

Policy information about studies with human participants or human data. See also policy information about sex, gender (identity/presentation), and sexual orientation and race, ethnicity and racism.

| | |
|---|---|
| Reporting on sex and gender | Sex and gender related analyses are not included in this study |
| Reporting on race, ethnicity, or other socially relevant groupings | We randomly selected households from the pilot areas to conduct a survey investigating the demographic characteristics of the household, such as whether they lived with children or elderly individuals. |
| Population characteristics | In this study, we collected data from 205,129 households to estimate the heterogeneity of reduce peak load when households are subject to incentive-based EDR policy during heatwave. The more detailed demographic information of the study participants (whether there are vulnerable groups, such as the elderly, children, etc.) is summarized in the "Supplementary Information". |
| Recruitment | We first adopted the clustered randomization method to randomly select the region of the EDR trial (divided by communities, a total of 205,129 households were selected) and then installed HPLC smart meters, which can collect electricity consumption information at 15-minute intervals. We randomly assigned permission to apply for the EDR trial and got three samples (sample A named EDR group, sample B named no-reply group, sample C named no-notification group), where sample C did not receive any EDR messages during the entire trial period. Before the experimental period began, we informed households who won the random assignment permission of how they were going to receive the treatments.<br>We randomly selected households to carry out the survey in the pilot area. The response rate (considering only valid responses) is 7774/10254 = 75.8%. Specifically, 10,254 questionnaires were distributed and 8,548 questionnaires were returned. We then tested the validity of the surveys through a rigorous screening process, which includes the following steps: 1). Dropped the error meter numbers that cannot be matched the electricity data. 2). The answer time had to be more than 60 seconds. 3). No more than 10% of the questionnaire items could be incomplete. 4). Questionnaires were excluded if the family member structure was unreasonable; for example, all family members were under the age of 18. Finally, we obtained 7,774 valid questionnaires. |
| Ethics oversight | The State Grid Institutional Review Board approved the experiments described in this article (including all trial households and survey). Informed consent was obtained from all the trial respondents when we started the trial. |

Note that full information on the approval of the study protocol must also be provided in the manuscript.

# Field-specific reporting

Please select the one below that is the best fit for your research. If you are not sure, read the appropriate sections before making your selection.

☐ Life sciences ☒ Behavioural & social sciences ☐ Ecological, evolutionary & environmental sciences

For a reference copy of the document with all sections, see nature.com/documents/nr-reporting-summary-flat.pdf

# Behavioural & social sciences study design

All studies must disclose on these points even when the disclosure is negative.

| | |
|---|---|
| Study description | Panel data analysis. We used quantitative methods for analysis, such as difference-in-difference(DID), difference-in-difference-in-difference(DDD) and ordinary least square(OLS) fixed effects regression.<br>In addition, we randomly selected households from the pilot areas to conduct a survey investigating the demographic characteristics of the household, such as whether they lived with children or elderly individuals, etc. |
| Research sample | We carried out this research with the state grid. We first adopted the clustered randomization method to randomly select the region of the EDR pilot (divided by communities, a total of 205,129 households were selected) and then installed HPLC smart meters, which can collect electricity consumption data at 15-minute intervals. We used the exact same procedures to conduct six EDR pilots based on monetary rewards in southwestern China from July 18, 2019, to August 21, 2019.<br>Our results are most credibly extended to other provinces with relatively similar climate and economic conditions, which cover |

| | |
|---|---|
| | roughly one-fifth of China's population and one-fifth of the gross domestic product.<br>Respondents participating in the survey must: 1) 18 years old or older, 2) understand the family's demographic attributes and income level, 3) understand or be responsible for the electricity bill of the house |
| Sampling strategy | The EDR policy was designed as a randomized control trial featuring three sample groups (each of which served a different purpose, as discussed below). We preprocessed the electricity use data (vacant homes that were always at 0 kWh were removed) and deleted the households with missing values caused by collection and transmission by HPLC smart meters.<br>Sample A (EDR group): The 16,072 households in this group installed an advanced HPLC meter and received the "EDR: economic incentives for energy conservation" message. Those in this group confirmed their participation.<br>Sample B (no-reply group): The 93,852 households in this group installed an advanced HPLC meter and received an "EDR: economic incentives for energy conservation" message. Those in this group did not confirm their participation.<br>Sample C (no-notification group): The 95,205 households in this group installed an advanced HPLC meter. Those in this group received no other treatment.<br>In addition, we used user_id-based sampling to randomly recruit households to participate in the survey. |
| Data collection | Electricity consumption data of households who have installed high-speed power line communication (HPLC) had been successfully collected every 15 minutes. |
| Timing | We collected 15-minute level electricity consumption data of households and the survey from July 18, 2019 to August 21, 2019. The cumulative file size is 5.11GB. |
| Data exclusions | We randomly selected households to carry out the survey in the pilot area. The response rate (considering only valid responses) is 7774/10254 = 75.8%. Specifically, 10,254 questionnaires were distributed and 8,548 questionnaires were returned. We then tested the validity of the surveys through a rigorous screening process, which includes the following steps: 1). Dropped the error meter numbers that cannot be matched the electricity data. 2). The answer time had to be more than 60 seconds. 3). No more than 10% of the questionnaire items could be incomplete. 4). Questionnaires were excluded if the family member structure was unreasonable; for example, all family members were under the age of 18. Finally, we obtained 7,774 valid questionnaires. |
| Non-participation | As descried in Sampling strategy, our main regression specifications leverage the random assignment to make comparisons between the treatment and control group. The random assignment supported permission to apply for the EDR rebate program. We can estimate the effect of random assignment selection by fitting difference-in-difference regressions and comparing the average outcome for all households selected in the random assignment (EDR group and no-reply group in our trial, which we called assignment winners) to the average outcome for all control households (no-notification, those not selected by the assignment). This is an intent-to-treat estimate. We can estimate the effect of EDR rebate coverage by fitting two-stage least squares regressions (with random assignment selection as an instrument for EDR rebate coverage) and estimating the local average treatment effect of EDR rebate coverage. Both approaches use the randomization of the assignment to estimate causal effects. |
| Randomization | We adopted the clustered randomization method to select regions for our EDR pilot and used user_id-based sampling to randomly recruit households to participate in the survey. |

# Reporting for specific materials, systems and methods

We require information from authors about some types of materials, experimental systems and methods used in many studies. Here, indicate whether each material, system or method listed is relevant to your study. If you are not sure if a list item applies to your research, read the appropriate section before selecting a response.

## Materials & experimental systems

| n/a | Involved in the study |
|---|---|
| ☒ | ☐ Antibodies |
| ☒ | ☐ Eukaryotic cell lines |
| ☒ | ☐ Palaeontology and archaeology |
| ☒ | ☐ Animals and other organisms |
| ☒ | ☐ Clinical data |
| ☒ | ☐ Dual use research of concern |
| ☒ | ☐ Plants |

## Methods

| n/a | Involved in the study |
|---|---|
| ☒ | ☐ ChIP-seq |
| ☒ | ☐ Flow cytometry |
| ☒ | ☐ MRI-based neuroimaging |
